# Supplementary material for: An apical ring protein essential for conoid complex assembly and daughter cell formation in Toxoplasma gondii
Source: Nat Commun. 2025 Nov 28;16:10149. doi: 10.1038/s41467-025-65382-y (PMC12663574; doi:10.1038/s41467-025-65382-y)
Supplement: Supplementary file 1 — Supplementary Information [file 41467_2025_65382_MOESM1_ESM.pdf]

Supplementary Information for

An apical ring protein essential for conoid complex assembly and daughter cell formation in *Toxoplasma gondii*

Wei Li[1,2*+], Oliwia Koczy[3,4*], Peipei Qin[2], Ignasi Forné[5], Simon Gras[2], Jennifer Grünert[6], Andreas Klingl[6], Simone Mattei[3,7], Elena Jimenez-Ruiz[2+] and Markus Meissner[2+]

* These authors contributed equally

\+ Corresponding authors: WL (weili93@sicau.edu.cn), EJR (elena.jimenez@para.vetmed.uni-muenchen.de) and MM (markus.meissner@para.vetmed.uni-muechen.de)

The PDF file includes:

Supplementary Figures

Supplementary Tables

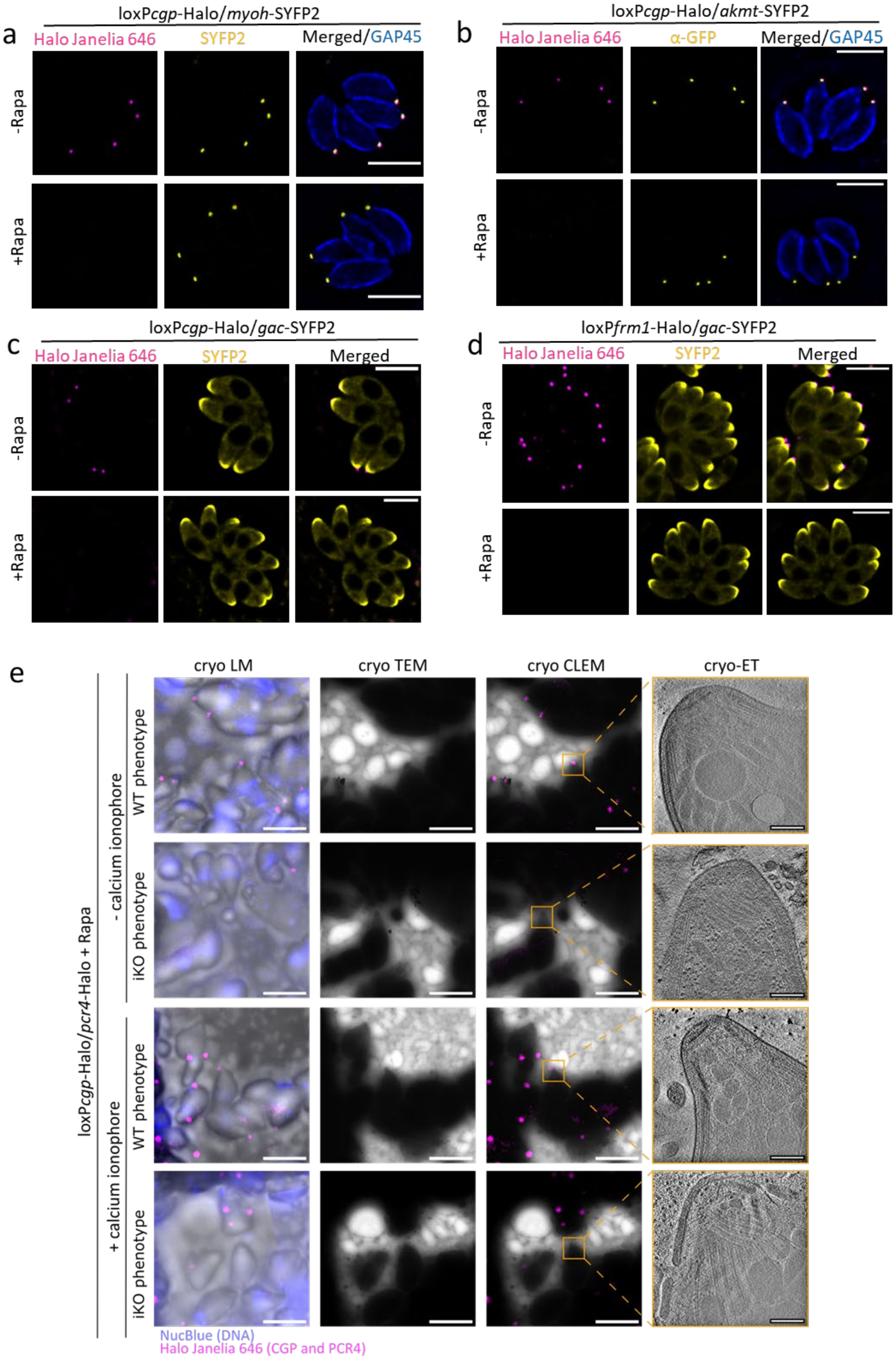

a
loxPcgp-Halo/myoh-SYFP2
Halo Janelia 646
SYFP2
Merged/GAP45
-Rapa
+Rapa
b
loxPcgp-Halo/akmt-SYFP2
Halo Janelia 646
α-GFP
Merged/GAP45
-Rapa
+Rapa
c
loxPcgp-Halo/gac-SYFP2
Halo Janelia 646
SYFP2
Merged
-Rapa
+Rapa
d
loxPfrm1-Halo/gac-SYFP2
Halo Janelia 646
SYFP2
Merged
-Rapa
+Rapa
e
cryo LM
cryo TEM
cryo CLEM
cryo-ET
loxPcgp-Halo/pcr4-Halo + Rapa
- calcium ionophore
+ calcium ionophore
WT phenotype
iKO phenotype
WT phenotype
iKO phenotype
NucBlue (DNA)
Halo Janelia 646 (CGP and PCR4)

Supplementary Fig. 1. Effect of CGP depletion on gliding motility related proteins and Cryogenic Corelative light and electron microscopy. a-d Analysis of CGP or FRM1 deletion on proteins localized to different sub-compartments of the conoid complex. Marker proteins were C-terminally tagged as indicated. Deletion of *cgp* or *frm1* was induced with rapamycin (Rapa) and imaging performed 72 hours later, either on fixed or live parasites, as indicated. Scale bars: 5 µm **a**, Effect of CGP depletion on MyoH (n=2 independent experiments). **b**, Effect of CGP depletion on AKMT (n=2 independent experiments). **c**, Effect of CGP depletion on GAC (n=2 independent experiments). This image was taken in live parasites. **d**, Effect of FRM1 depletion on GAC (n=2 independent experiments). This image was taken in live parasites. **e**, Cryogenic Corelative light and electron microscopy (cryoCLEM) allows to distinguish between WT and iKO parasites (n=2 independent experiments). Column 1: cryogenic light microscopy (cryo LM) of WT and iKO parasites, magenta – CGP and Pcr4 (Halo Janelia 646), blue – DNA (NucBlue). Column 2: cryogenic transmission electron microscopy (cryo TEM) of the same field of view as in column 1. Column 3: Correlation of the images from light and electron microscopy. Column 4: Slice of a tomographic reconstruction of the parasites marked with orange squares in column 3. Scale bar 5 µm columns 1, 2, 3; scale bar 200 nm column 4.

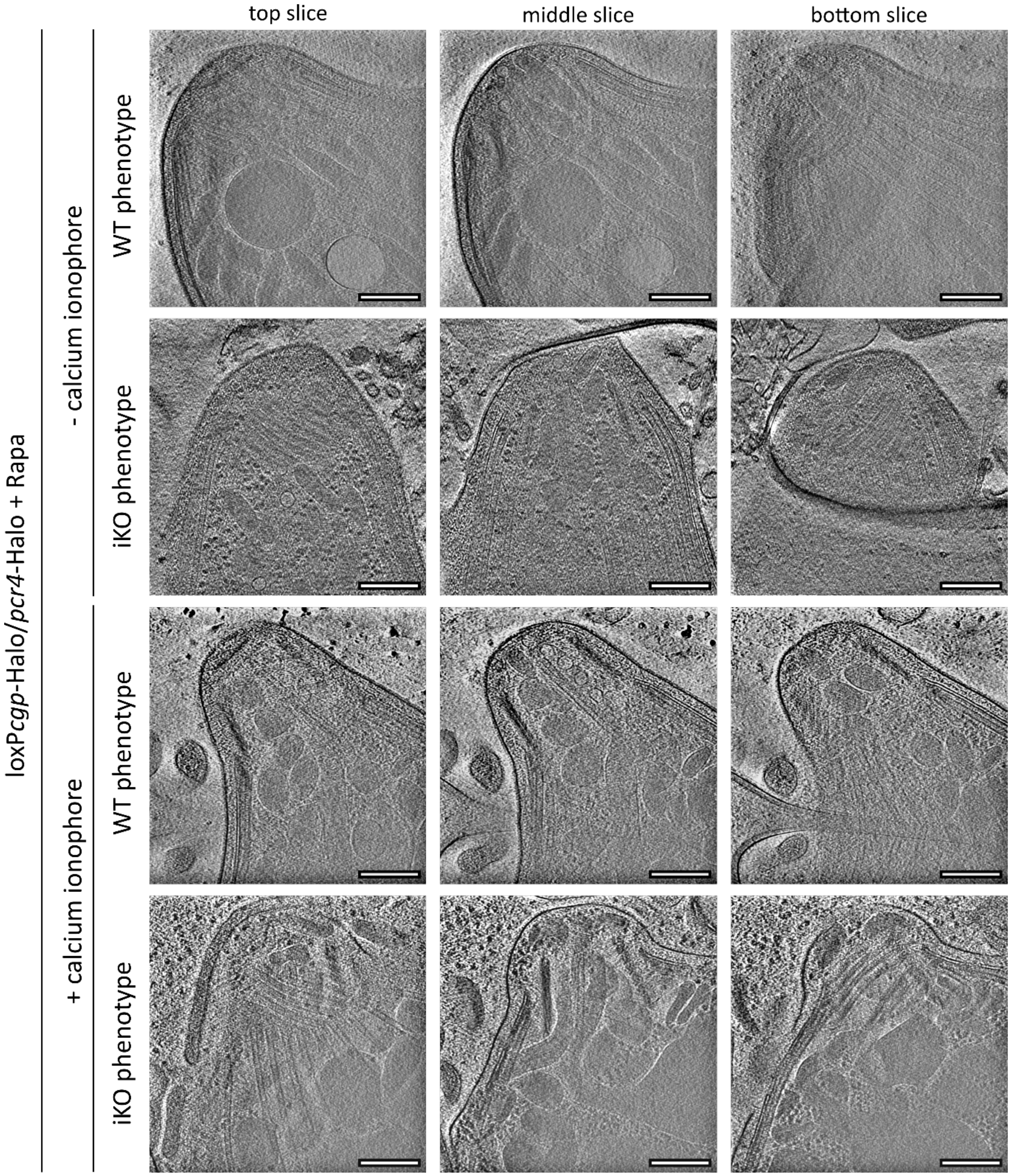

Supplementary Fig. 2. CGP depleted parasites are deprived of the preconoidal rings. Example slices of tomographic reconstruction of WT and *cgp* iKO phenotype parasites as in Fig. 2c without the highlights of the structural components. First row – WT phenotype parasite without calcium ionophore treatment, second row – iKO phenotype parasite without calcium ionophore treatment, third row – WT phenotype parasite after calcium ionophore treatment, fourth row – iKO phenotype parasite after calcium ionophore treatment. n = 2 independent experiments. Scale bars 200 nm.

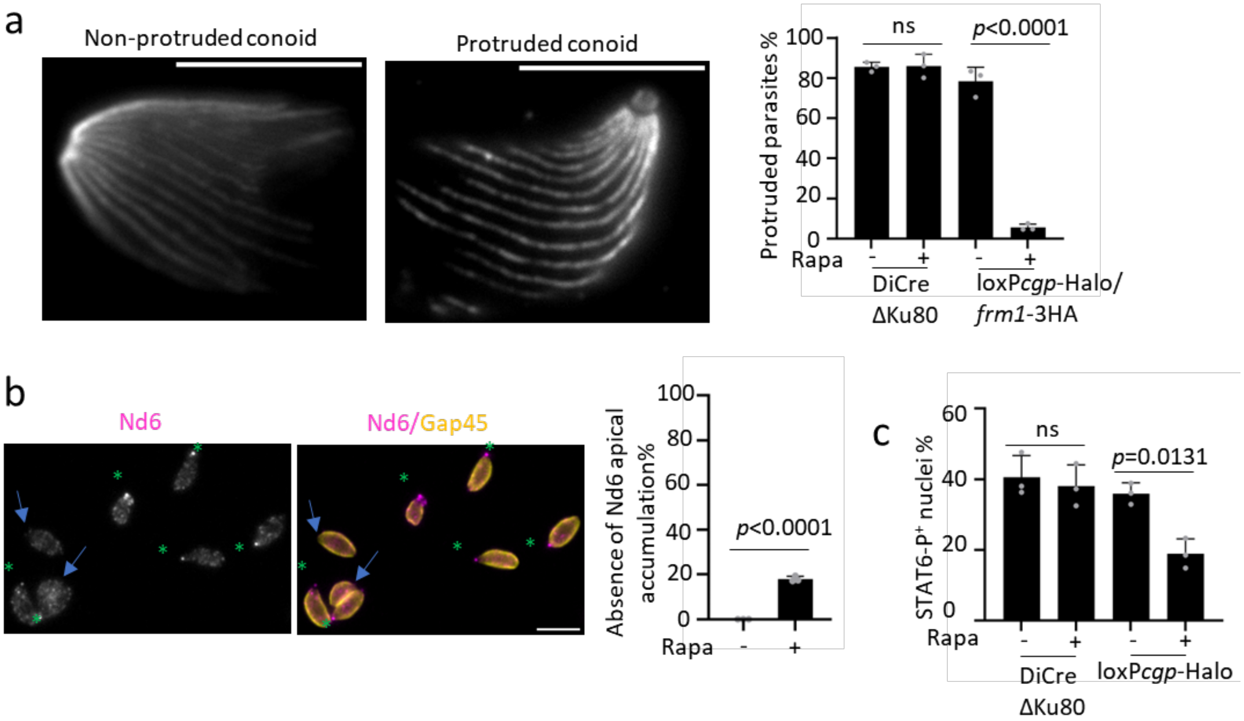

Supplementary Fig. 3. CGP depletion causes conoid protrusion defects, loss of apical Nd6 localisation in a subset of parasites, and impaired rhoptry secretion. a, ExM images showing examples of protruded and non-protruded conoids via acetylated tubulin (AcTub) staining, along with quantification of conoid protrusion in the indicated parasite lines with or without rapamycin treatment (n=3 independent experiments). Effects were assessed 72 hours post-induction. Data are presented as mean ± s.d. Statistical analysis was performed using two-sided one-way ANOVA with Tukey's test (multiple-comparison adjusted). Ns, non-significant. Scale bar: 10 µm. **b,** images showing Nd6 staining (magenta) and GAP45 staining (yellow) in loxP*cgp*-Halo/*nd6*-SYFP2 parasites, along with quantification following 72 hours of rapamycin treatment (n=3 independent experiments). Blue arrows indicate the loss of apical Nd6 accumulation. Green asterisks indicate the presence of apical Nd6 signals. Data are presented as mean ± s.d. Statistical significance was determined using an unpaired two-tailed Student's *t*-test. Scale bar: 5 µm. **c,** Quantification of phosphorylated STAT6-positive nuclei in the indicated parasite lines with or without rapamycin treatment for 72 hours (n=3 independent experiments). Data are presented as mean ± s.d. Two sided one-way ANOVA with Tukey's test (multiple-comparison adjusted).

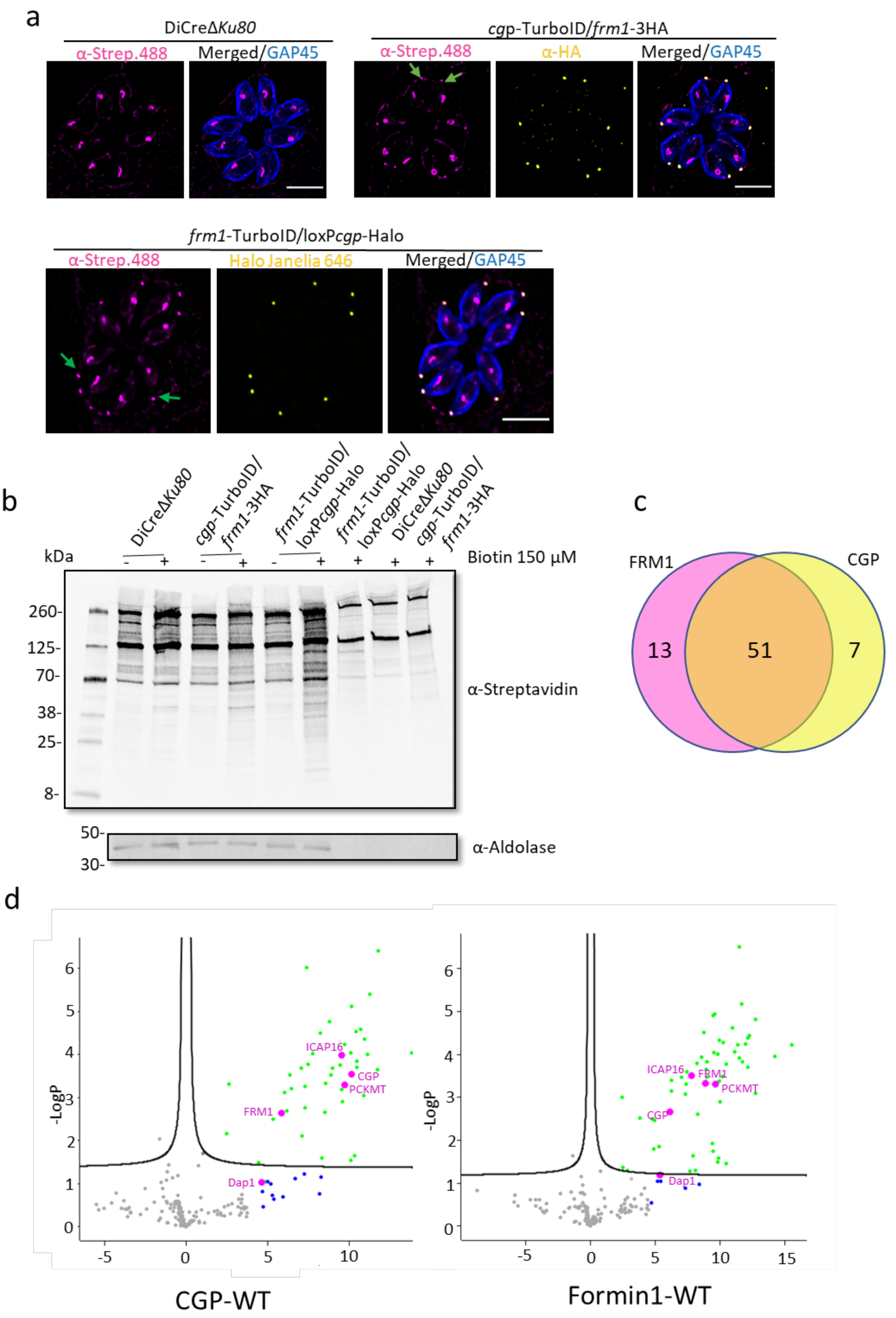
a
DiCreΔKu80
α-Strep.488
Merged/GAP45
cgp-TurboID/frm1-3HA
α-Strep.488
α-HA
Merged/GAP45
frm1-TurboID/loxPcgp-Halo
α-Strep.488
Halo Janelia 646
Merged/GAP45
b
DiCreΔKu80
cgp-TurboID/ frm1-3HA
frm1-TurboID/ loxPcgp-Halo
frm1-TurboID/ loxPcgp-Halo
DiCreΔKu80
cgp-TurboID/ frm1-3HA
kDa
Biotin 150 µM
- + - + - + + + +
260-
125-
70-
38-
25-
8-
α-Streptavidin
50-
30-
α-Aldolase
c
FRM1
CGP
13
51
7
d
-LogP
ICAP16
CGP
PCKMT
FRM1
Dap1
CGP-WT
ICAP16
FRM1
PCKMT
CGP
Dap1
Formin1-WT

Supplementary Fig. 4. Identification of interactors by BioID. a, IFA of WT and TurboID-expressing parasites stained with Alexa Fluor 488 Streptavidin (α-Strep.488) and corresponding antibodies or dyes for the bait proteins (n=2 independent experiments). Green arrows indicate biotinylated proteins at the apical tip. Scale bar: 5 µm. **b**, WB showing biotinylated proteins in the indicated parasite lines after 24 hours of culture, followed by 6 hours of 150 µM biotin treatment (n=3 independent experiments). Parasite pellets were lysed and tested by Western Blot. The last three lanes show proteins eluted from streptavidin beads. Anti-aldolase was used as a loading control. **c**, Venn diagram illustrating the overlap in gene numbers that are highly or significantly enriched in the FRM1-TurboID and CGP-TurboID datasets (n=3 independent experiments). High enrichment was defined as a greater than 4.5-fold increase in protein abundance in TurboID-tagged parasites relative to WT controls. **d,** Volcano plots showing the relative enrichment of CGP (left panel) and FRM1 (right panel), defined as the value of protein enrichment in TurboID-tagged parasite lines minus that in WT parasites. N=3 independent experiments. Significant hits are green; highly enriched (over 4.5) but not significant hits are blue. Proteins absent in *cgp* KO are shown in magenta.

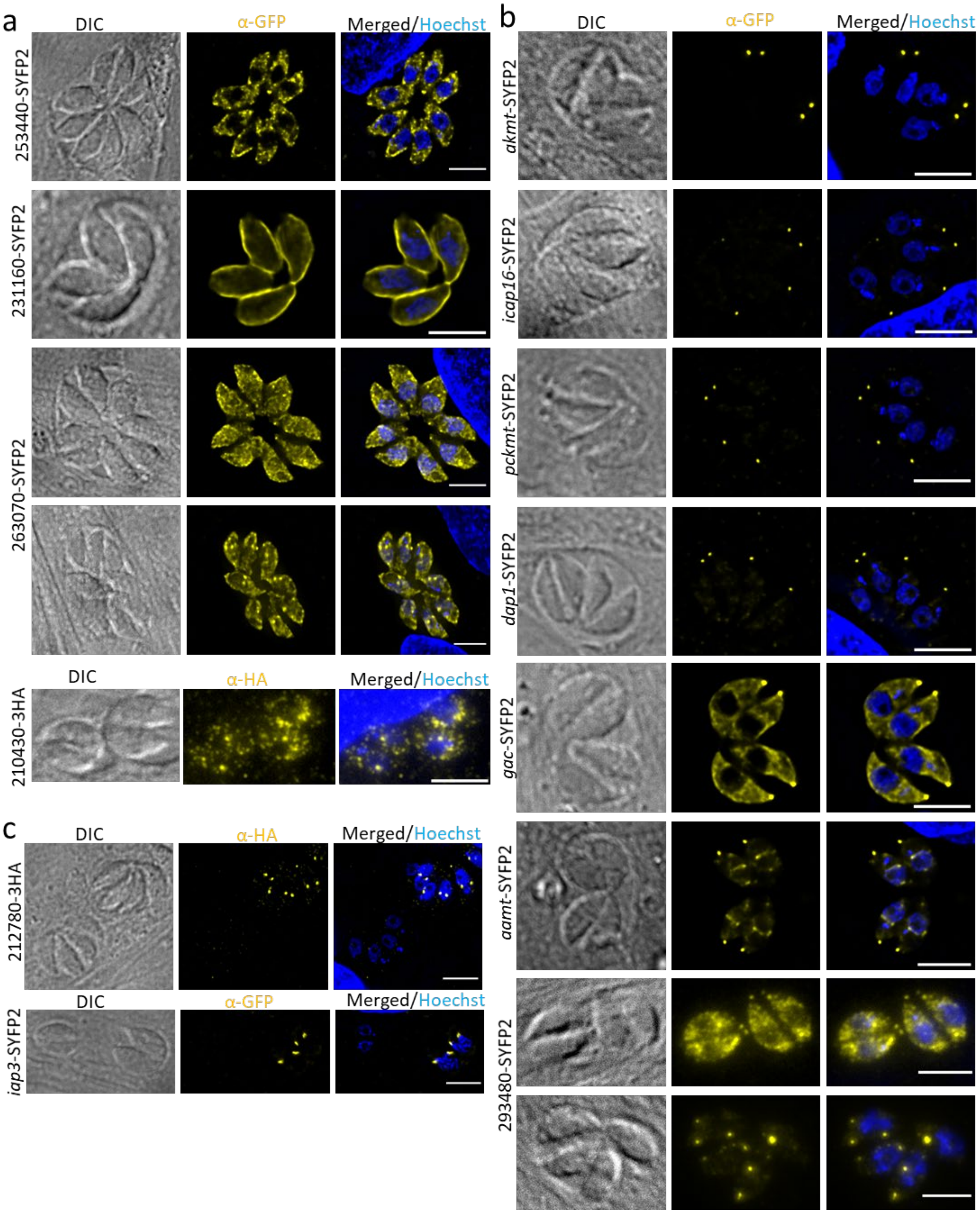

Supplementary Fig. 5. Localization of selected candidate proteins from the proximity labelling experiments. All proteins were C-terminally tagged as indicated**.** N = 2 independent experiments. **a**, Proteins showing non-conoid complex localization. Scale bar: 5 µm. **b**, Proteins (partially) localized at the parasite apex. Scale bar: 5 µm. **c**, Proteins with cell cycle–dependent signal. Scale bar: 5 µm.

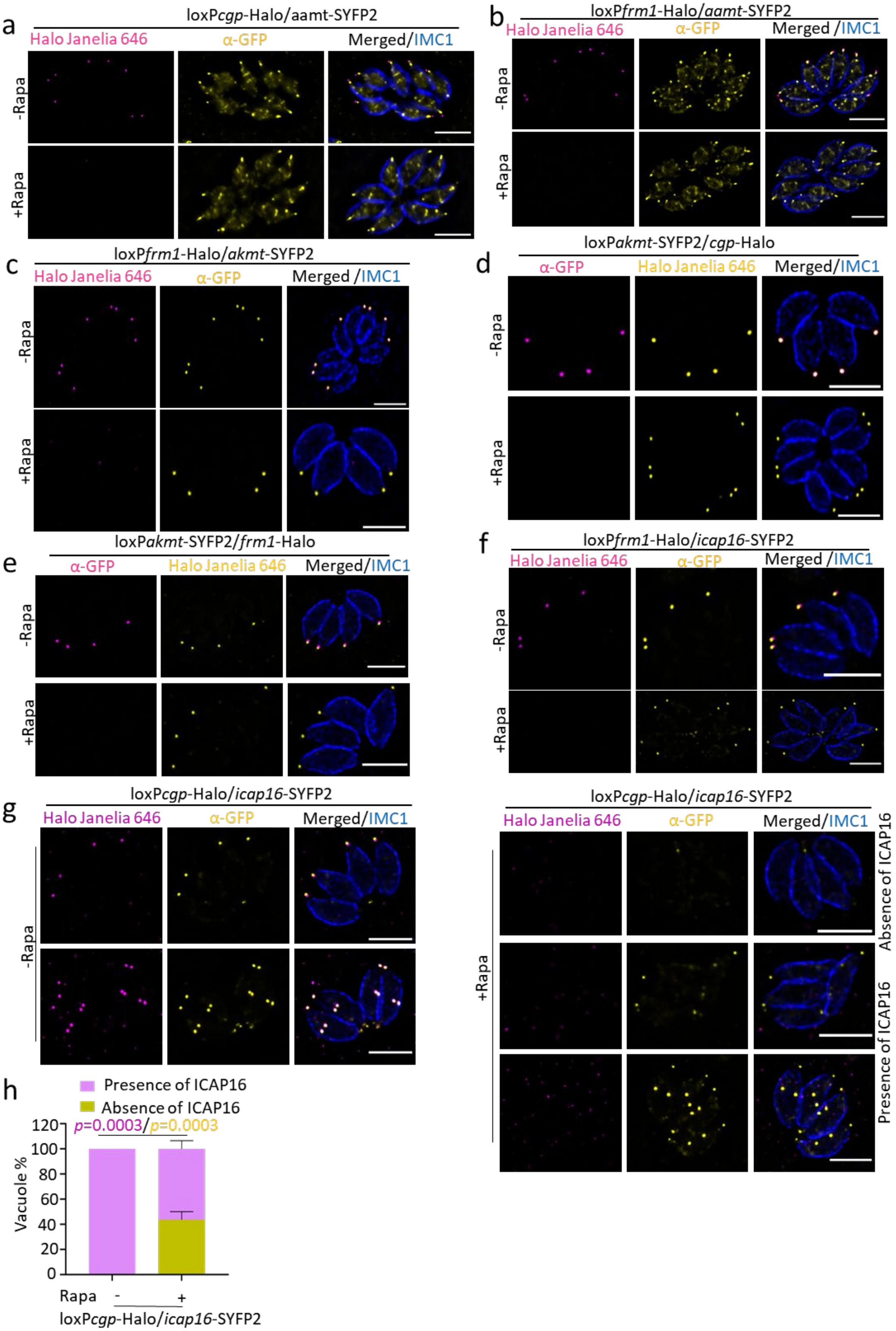
a
loxPcgp-Halo/aamt-SYFP2
Halo Janelia 646
α-GFP
Merged/IMC1
-Rapa
+Rapa
b
loxPfrm1-Halo/aamt-SYFP2
Halo Janelia 646
α-GFP
Merged /IMC1
-Rapa
+Rapa
c
loxPfrm1-Halo/akmt-SYFP2
Halo Janelia 646
α-GFP
Merged /IMC1
-Rapa
+Rapa
d
loxPakmt-SYFP2/cgp-Halo
α-GFP
Halo Janelia 646
Merged/IMC1
-Rapa
+Rapa
e
loxPakmt-SYFP2/frm1-Halo
α-GFP
Halo Janelia 646
Merged/IMC1
-Rapa
+Rapa
f
loxPfrm1-Halo/icap16-SYFP2
Halo Janelia 646
α-GFP
Merged /IMC1
-Rapa
+Rapa
g
loxPcgp-Halo/icap16-SYFP2
Halo Janelia 646
α-GFP
Merged/IMC1
-Rapa
loxPcgp-Halo/icap16-SYFP2
Halo Janelia 646
α-GFP
Merged/IMC1
+Rapa
Absence of ICAP16
Presence of ICAP16
h
Presence of ICAP16
Absence of ICAP16
p=0.0003/p=0.0003
Vacuole %
120
100
80
60
40
20
0
Rapa
-
+
loxPcgp-Halo/icap16-SYFP2

Supplementary Fig. 6. Depletion of CGP leads to the absence of PCR proteins but not other apical proteins. All proteins were C-terminally tagged in the respective iKOs as indicated**. a**, Depletion of CGP did not affect AAMT localization (n=2 independent experiments). **b**, Depletion of FRM1 did not affect AAMT localization (n=2 independent experiments). **c**, Depletion of FRM1 did not affect AKMT localization (n=2 independent experiments). **d**, Depletion of AKMT did not impact CGP localization (n=2 independent experiments). **e**, Depletion of AKMT did not impact FRM1 localization (n=2 independent experiments). **f**, Depletion of FRM1 did not affect ICAP16 localization (n=2 independent experiments). **g**, Representative images of ICAP16 localization (presence and absence) upon CGP depletion (n=3 independent experiments). **h**, Quantification of (g). Data are presented as mean ± s.d. *p*-values were calculated using a two-tailed unpaired Student's *t*-test.

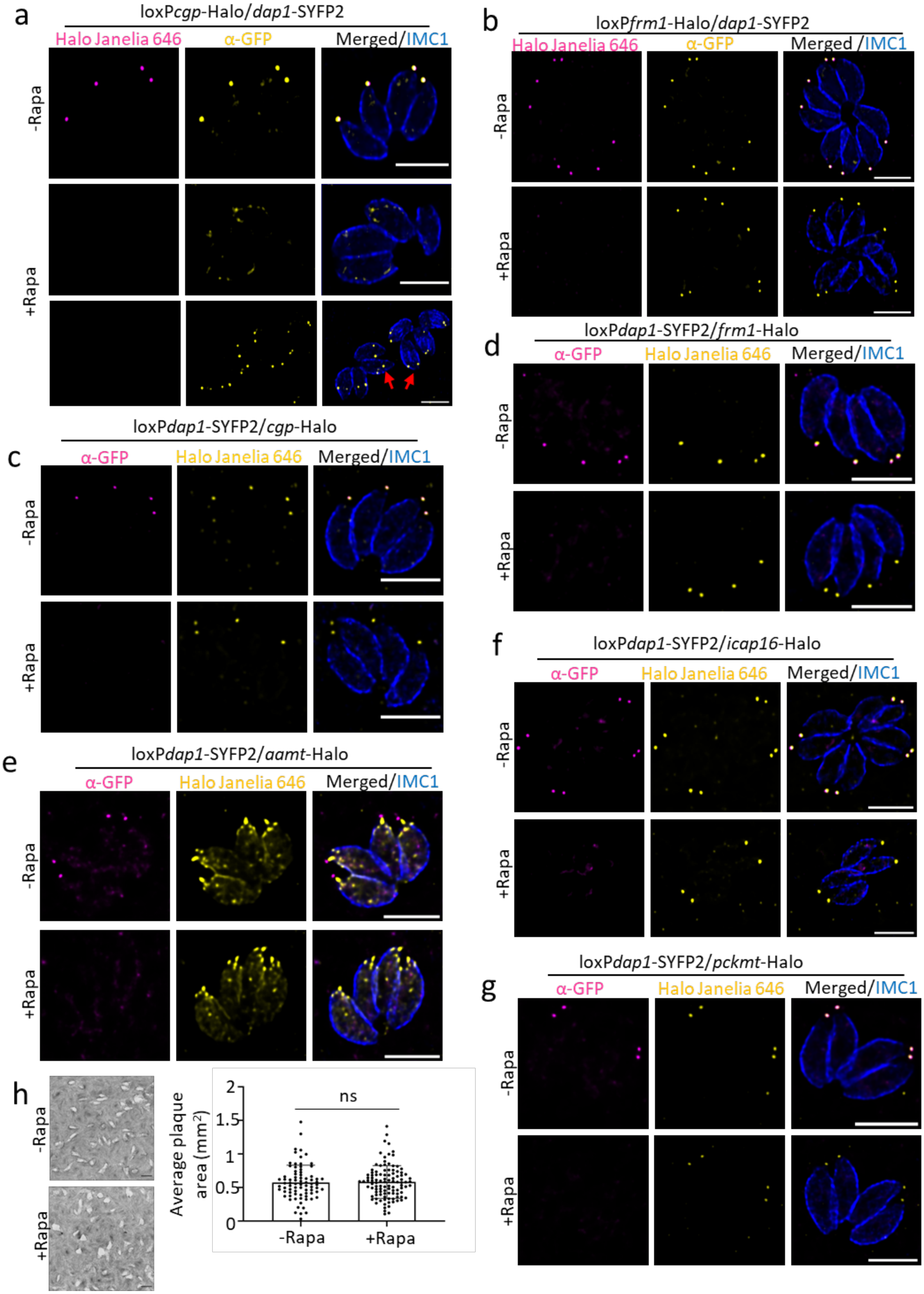
a
loxPcgp-Halo/dap1-SYFP2
Halo Janelia 646
α-GFP
Merged/IMC1
-Rapa
+Rapa
b
loxPfrm1-Halo/dap1-SYFP2
Halo Janelia 646
α-GFP
Merged /IMC1
-Rapa
+Rapa
c
loxPdap1-SYFP2/cgp-Halo
α-GFP
Halo Janelia 646
Merged/IMC1
-Rapa
+Rapa
d
loxPdap1-SYFP2/frm1-Halo
α-GFP
Halo Janelia 646
Merged/IMC1
-Rapa
+Rapa
e
loxPdap1-SYFP2/aamt-Halo
α-GFP
Halo Janelia 646
Merged/IMC1
-Rapa
+Rapa
f
loxPdap1-SYFP2/icap16-Halo
α-GFP
Halo Janelia 646
Merged/IMC1
-Rapa
+Rapa
g
loxPdap1-SYFP2/pckmt-Halo
α-GFP
Halo Janelia 646
Merged/IMC1
-Rapa
+Rapa
h
-Rapa
+Rapa
Average plaque area (mm2)
2
1.5
1
0.5
0
ns
-Rapa
+Rapa

Supplementary Fig. 7. Dap1 is not essential for the parasite lytic cycle. All proteins were C-terminally tagged in the respective iKOs as indicated. **a**, Depletion of CGP led to the loss of Dap1 in mature PCRs but not in daughter cells (red arrows). N=2 independent experiments. Scale bar: 5 µm. **b**, Depletion of FRM1 did not affect Dap1 localization (n=2 independent experiments). Scale bar: 5 µm. **c-g**, Localization of CGP (**c**), FRM1 (**d**), AAMT (**e**), ICAP16 (**f**), and PCKMT (**g**) were unaffected upon Dap1 depletion (n=2 independent experiments). Scale bar: 5 µm. **h**, Plaque assays and quantification of plaque size in loxP*dap1*-SYFP2 parasites, with or without rapamycin treatment (n=3 independent experiments). Scale bar: 1000 µm. Data are presented as mean ± s.d. *P*-values were calculated using a two-tailed unpaired Student's t-test. Ns, non-significant.

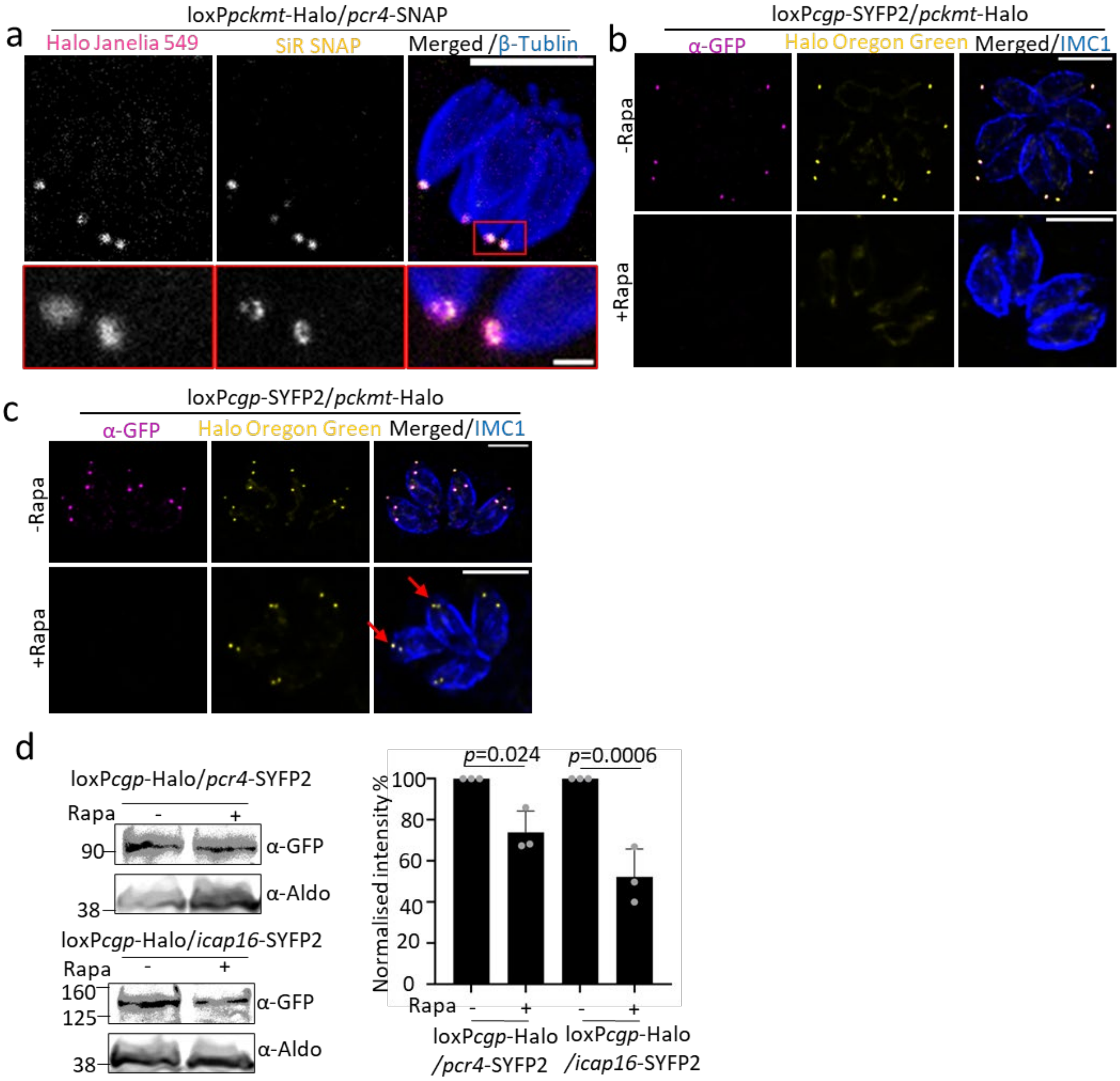

Supplementary Fig. 8. CGP depletion leads to the absence of the PCR protein PCKMT and western blot analysis of the indicated parasites. All proteins were C-terminally tagged in the respective iKOs as indicated. **a,** Colocalization analysis of PCKMT and Pcr4. Confocal imaging (upper panel) and STED imaging (lower panel) show PCKMT colocalized with Pcr4. Lower panel shows STED images of the boxed area. N=2 independent experiments. Scale bars: upper panel, 5 µm; lower panel, 0.5 µm. **b,** CGP depletion resulted in the loss of PCKMT in mature cells. N=2 independent experiments. **c,** CGP depletion resulted in the absence of PCKMT in mature PCRs but not daughter PCRs (red arrows). N=2 independent experiments. Scale bar: 5 µm. **d,** Effect of CGP depletion on Pcr4 and ICAP16 protein levels. Western blot analysis of the loxP*cgp*-Halo/*pcr4*-SYFP2 and loxP*cgp*-Halo/*icap16*-SYFP2 parasites pretreated with or without rapamycin for 72 hours and subjected to Western blot analysis. Left panel: representative western blot images. Aldolase (Aldo) was used as loading control. Protein marker size is indicated on the left. Right panel: quantification of normalized intensity for Pcr4 and ICAP16. Each protein intensity was first

normalized to Aldolase and then to the minus Rapamycin condition. N=3 independent experiments. Data are presented as mean ± s.d. *P*-values were calculated using two-sided one-way ANOVA with Tukey's test (multiple-comparison adjusted).

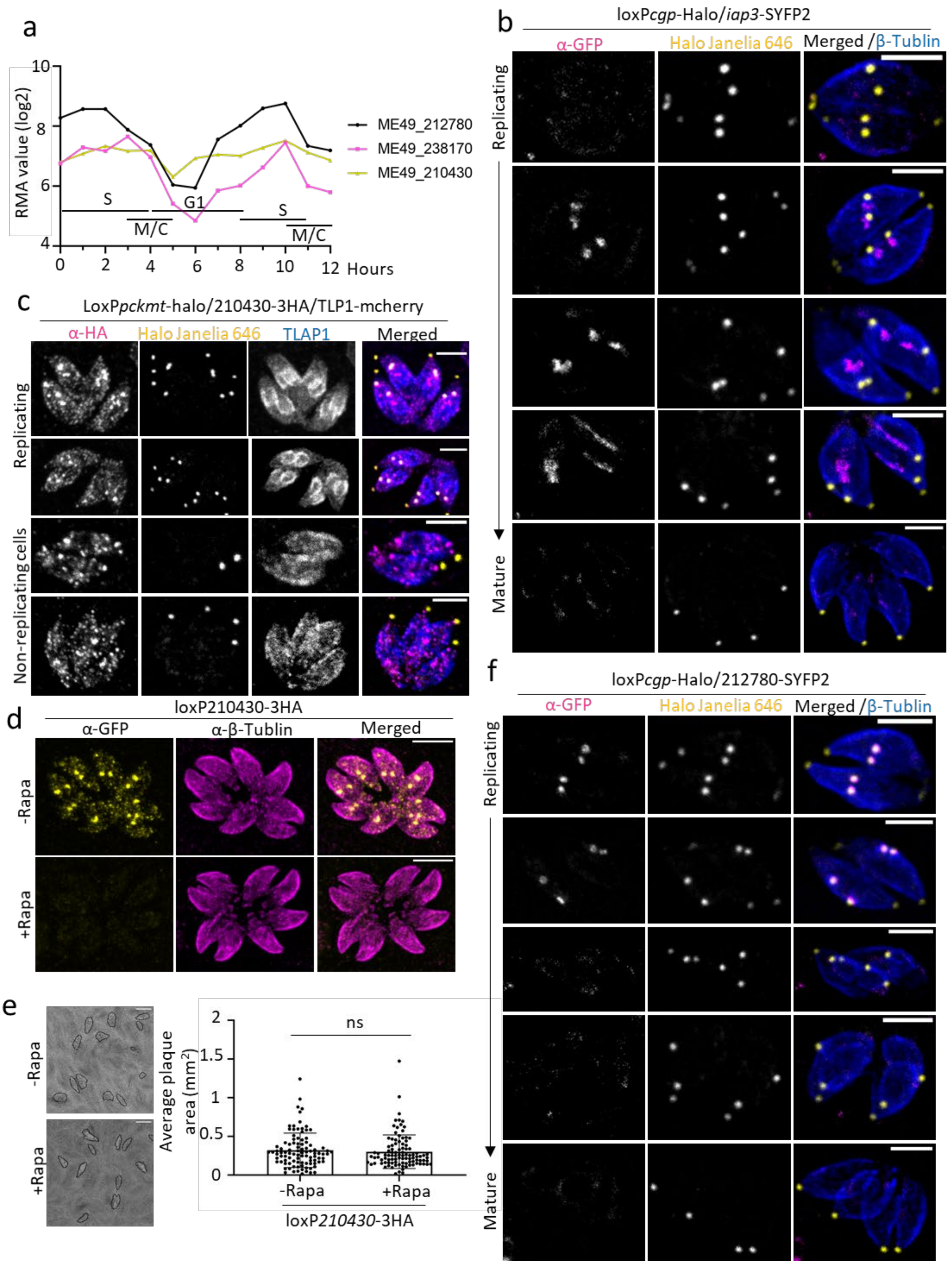
a
RMA value (log2)
Hours
ME49_212780
ME49_238170
ME49_210430
S
G1
M/C
b
loxPcgp-Halo/iap3-SYFP2
α-GFP
Halo Janelia 646
Merged /β-Tublin
Replicating
Mature
c
LoxPpckmt-halo/210430-3HA/TLP1-mcherry
α-HA
Halo Janelia 646
TLAP1
Merged
Replicating
Non-replicating cells
d
loxP210430-3HA
α-GFP
α-β-Tublin
Merged
-Rapa
+Rapa
e
Average plaque area (mm²)
ns
loxP210430-3HA
f
loxPcgp-Halo/212780-SYFP2
α-GFP
Halo Janelia 646
Merged /β-Tublin
Replicating
Mature

Supplementary Fig. 9. Identification of daughter cell-specific proteins. All proteins were C-terminally tagged in the respective iKOs as indicated. **a,** Cell cycle expression profile with indicated proteins selected from the proximity labelling assays. **b,** Confocal images show that CGP does not associate with IAP3 (TGGT1_238170) during the cell cycle. N=2 independent experiments. Scale bar: 5 µm. **c,** TGGT1_210430 is expressed throughout the cell cycle and shows some colocalisation with the PCR protein, PCKMT, in replicating stage. N=2 independent experiments. Scale bar: 5 µm. TLAP1 (TrxL1-associating proteins)[51] endogenously tagged was used to indicate parasites in replicating and non-replicating stages. **d,** Confocal images show depletion of TGGT1_210430 at 48 hours post-induction with rapamycin. N=2 independent experiments. Scale bar: 5 µm. **e,** Plaque assay of loxP210430 parasites shows no impact on plaque formation upon TGGT1_210430 depletion. Plaques are highlighted in black. Plaque size were measured and are shown in the bar graph. N=3 independent experiments. Scale bar: 1000 µm. Data are presented as mean ± s.d. *P*-values were calculated using a two-tailed unpaired Student's *t*-test. Ns, non-significant. **f,** Confocal images showing ASAF1 expression during different stages of endodyogeny. ASAF1 shows good colocalization with CGP until ASAF1 becomes undetectable in the later stages of endodyogeny (see also Fig. 3b). N=2 independent experiments. Scale bar: 5 µm.

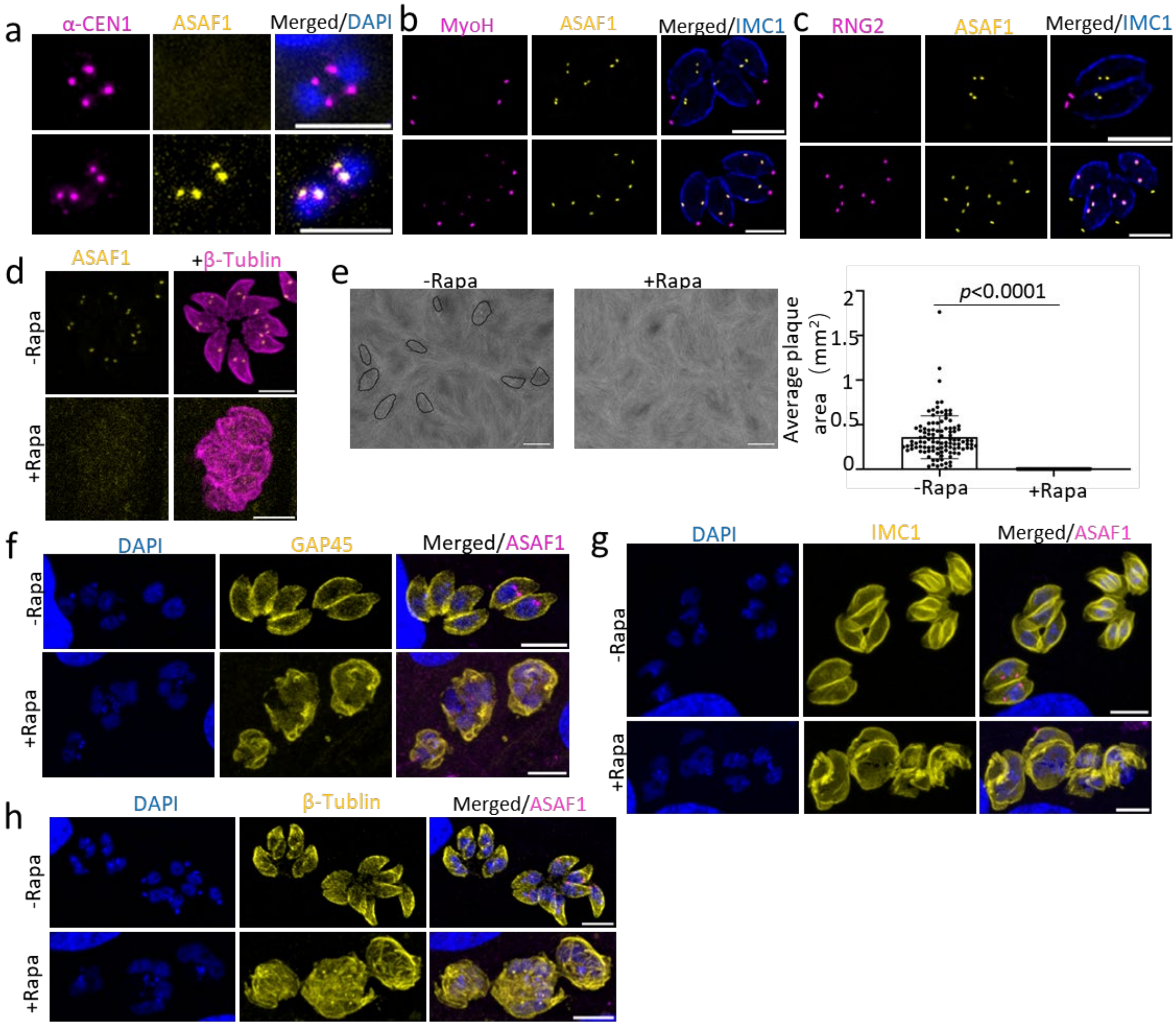

Supplementary Fig. 10. ASAF1 is recruited at very early endodyogeny and is an essential for protein. a, Centrin1 was used to detect the centrosome. ASAF1 is detected after Centrin 1 duplication. N=2 independent experiments. Scale bar: 3 µm. **b,** ASAF1 is detected before MyoH appears recruited in daughter conoid complex (top images). At a later stage ASAF1 and MyoH show good co-localisation. N=2 independent experiments. Scale bar: 5 µm. **c,** ASAF1 is detected before RNG2 is assembled in daughter conoid complex (top images). At a later stage, ASAF1 and RNG2 show good co-localisation. N=2 independent experiments. Scale bar: 5 µm. **d,** Confocal images showing ASAF1 depletion 48 hours post-induction with rapamycin in loxP*asaf1*-SYFP2. N=2 independent experiments. **e,** Plaque assay of loxP*asaf1*-SYFP2 strains shows that parasites lacking ASAF1 are unable to form plaques. Plaques are highlighted in black. Plaque size was measured and are shown in the bar graph. N=3 independent experiments. Scale bar: 1000 µm. Data are presented as mean ± s.d. *P*-values were calculated using a two-tailed unpaired Student's *t*-test. **f,** Confocal images showing the effect of ASAF1 depletion on GAP45 in the loxP*asaf1*-SYFP2 strain, 48 hours after induction with rapamycin. N=2 independent experiments. Scale bar: 5 µm. **g,** Confocal images showing the effect of ASAF1 depletion on IMC1 using the loxP*asaf1*-SYFP2

strain, 48 hours after induction with rapamycin. N=2 independent experiments. Scale bar: 5 µm. **h,** Confocal images showing the effect of ASAF1 depletion on microtubules using the loxP*asaf1*-SYFP2 strain, 48 hours after induction with rapamycin. N=2 independent experiments. Scale bar: 5 µm.

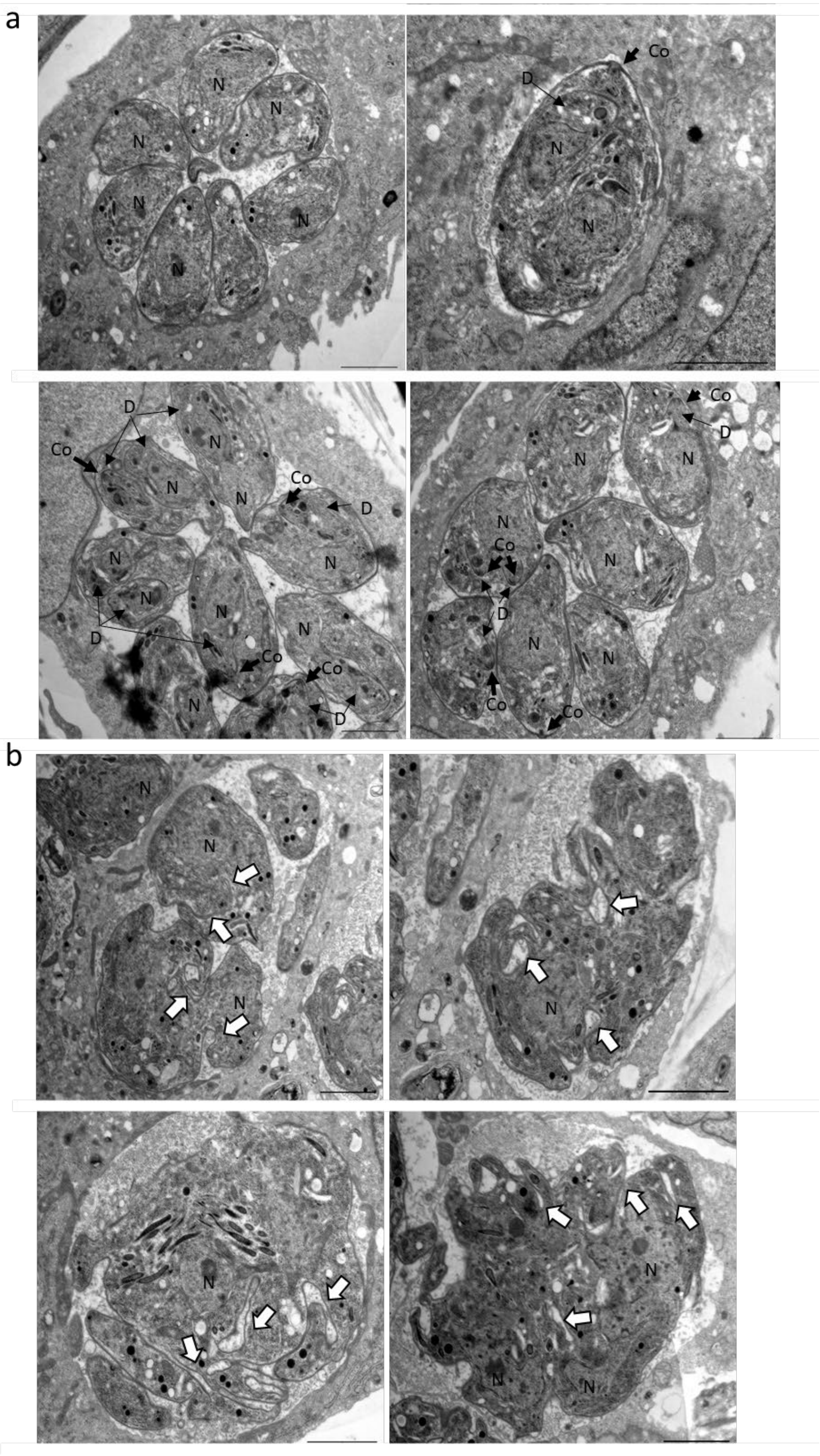
a
N
Co
D
b

Supplementary Fig. 11. TEM images of LoxP*asaf1*-SYFP2 non-induced and induced parasites. Additional examples of TEM images showing ultrastructural features of loxP*asaf1*-SYFP2 parasites under different conditions. a, TEM of non-induced LoxP*asaf1*-SYFP2 parasites. b, TEM on the induced LoxP*asaf1*-SYFP2 parasites after 48 hours rapamycin induction. Co: Conoid; D: daughter cell; N: Nuclei. Scale bar: 2 µm.

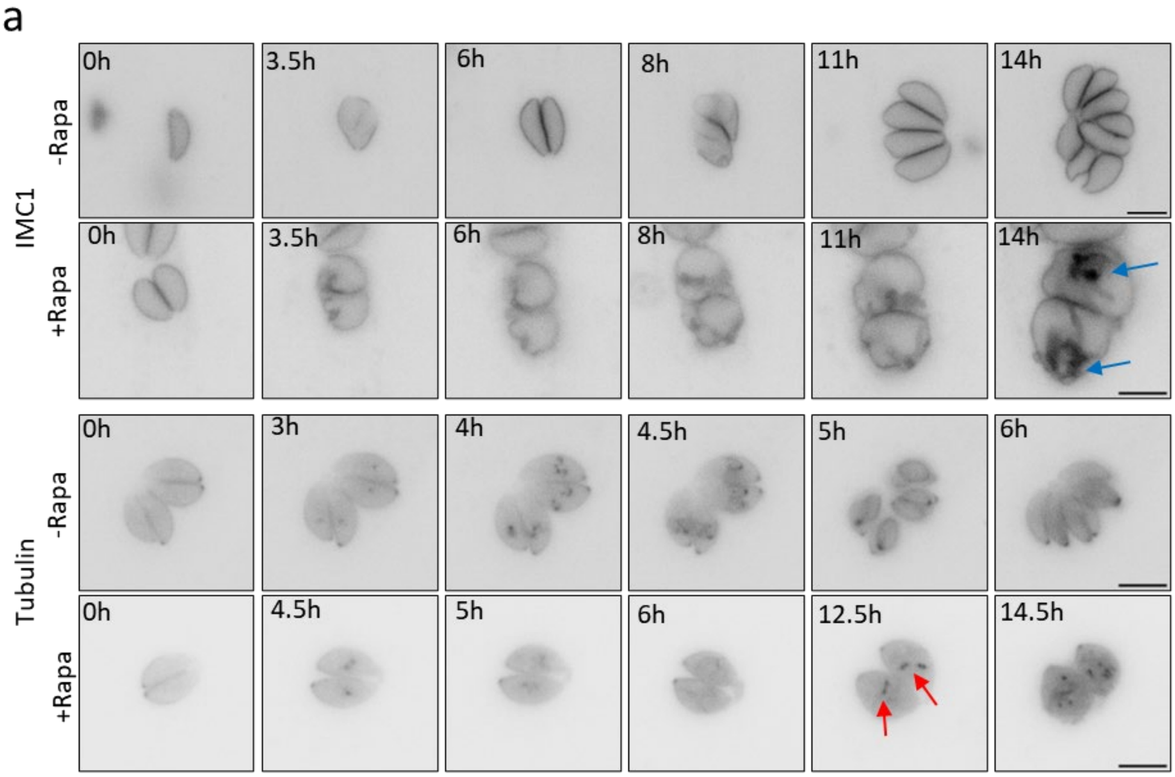

Supplementary Fig. 12, Time-lapse analysis of ASAF1 depletion on IMC and tubulin formation. loxP*asaf1*-SYFP2 parasites expressing IMC1-mCherry or transiently transfected with mCherry-Tubulin were allowed to express overnight before rapamycin was added to induce *asaf1* gene excision. Imaging started approximately 8 hours post-induction, with frames captured every 30 minutes. Selected frames highlight defects in daughter cell formation. Blue arrows indicate membrane folding. Note that spindle microtubules (red arrows) are still formed, but no formation of daughter buds can be detected. Images correspond to Supplementary Movie 7. Scale bar: 5 µm. At least N=3 biologically independent experiments.

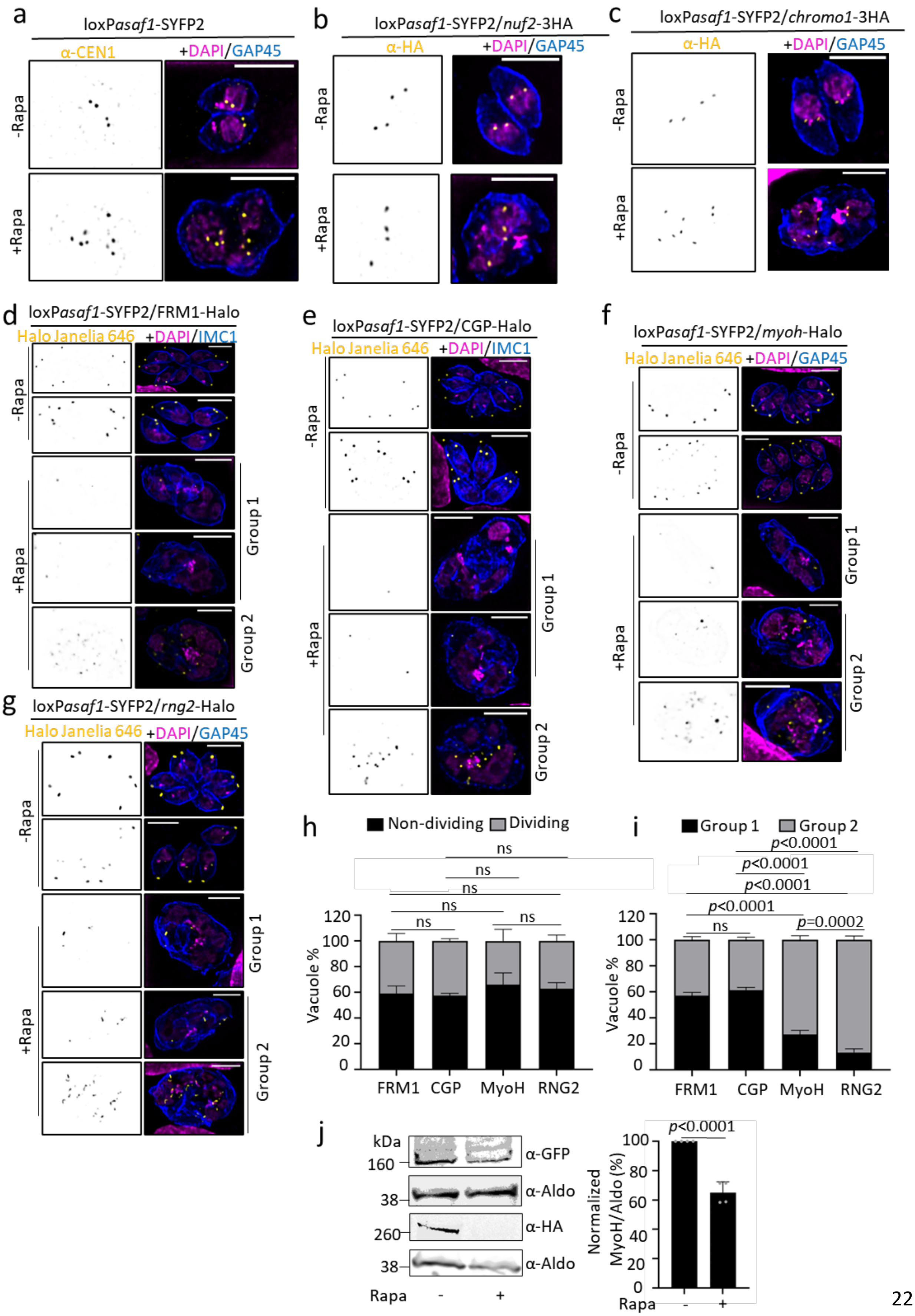

a
loxPasaf1-SYFP2
α-CEN1
+DAPI/GAP45
-Rapa
+Rapa
b
loxPasaf1-SYFP2/nuf2-3HA
α-HA
+DAPI/GAP45
c
loxPasaf1-SYFP2/chromo1-3HA
α-HA
+DAPI/GAP45
d
loxPasaf1-SYFP2/FRM1-Halo
Halo Janelia 646 +DAPI/IMC1
Group 1
Group 2
e
loxPasaf1-SYFP2/CGP-Halo
Halo Janelia 646 +DAPI/IMC1
f
loxPasaf1-SYFP2/myoh-Halo
Halo Janelia 646 +DAPI/GAP45
g
loxPasaf1-SYFP2/rng2-Halo
Halo Janelia 646 +DAPI/GAP45
h
Non-dividing
Dividing
ns
Vacuole %
FRM1
CGP
MyoH
RNG2
i
Group 1
Group 2
p<0.0001
p=0.0002
j
kDa
160
38
260
α-GFP
α-Aldo
α-HA
Rapa
Normalized MyoH/Aldo (%)

Supplementary Fig. 13. ASAF1 depletion does not affect general replication factors but impacts conoid complex proteins. All proteins were C-terminally tagged in the respective iKOs as indicated. **a,** Centrin 1 (CEN1) still divides and is associated with the nucleus. 1. N=2 independent experiments. Scale bar: 5 µm. **b,** Nuf2 still divides and is associated with the nucleus. Scale bar: 5 µm. **c,** Chomo1 still divides and is associated with the nucleus. Scale bar: 5 µm. **d,** Effect of ASAF1 depletion on FRM1. N=2 independent experiments.Scale bar: 5 µm. **e,** Effect of ASAF1 depletion on CGP. N=2 independent experiments. Scale bar: 5 µm. **f,** Effect of ASAF1 depletion on MyoH. N=2 independent experiments.Scale bar: 5 µm. **g,** Effect of ASAF1 depletion on RNG2. N=2 independent experiments. Scale bar: 5 µm. **h,** Quantification of vacuoles containing non-dividing and dividing parasite in non-induced KO parasites from (**d**-**g**). **i,** Quantification of vacuoles in induced KO parasites from (**d**-**g**). Group 1: Parasite vacuoles have 3 or fewer dots. Group 2: Parasite vacuoles exhibit multiple dotted signals (more than 3). Data presented as Mean ± SD. Statistics: One-Way ANOVA and Tukey's tests were employed. Ns, non-significant. **j,** Effect of ASAF1 depletion MyoH protein level. Western blot analysis of the loxP*asaf1*-HA/*myoh*-SYFP2 parasites pretreated with or without rapamycin for 48 hours and subjected to Western blot analysis. Left panel: representative western blot images. Aldolase (Aldo) was used as loading control. Protein marker size is indicated on the left. Right panel: quantification of normalized intensity for MyoH. MyoH protein intensity was first normalized to Aldolase and then to the minus rapamycin condition. Data are presented as mean ± s.d. *P*-values were calculated using a two-tailed unpaired Student's t-test.

Table S1. Summary of selected candidates identified in proximity labeling exeriments

Gene ID	Product	Phenotypic score	hyperLOPIT predicted localisation				Known localisation	Reference for localisation	Enrichment		Sucessful tagging in this tudy
			Final probability	TAGM-MAP	TAGM-MCMC	Top probability			CGP	Formin 1	
TGGT1_240380	CGP	-3.85	0	PM - peripheral 2	PM - peripheral 2	0.996	Preconidal ring	24	✓	✓	✓
TGGT1_462965	Formin 1	-2.8	N/A	N/A	N/A	N/A	Preconidal ring	5	✓	✓	✓
TGGT1_292170	PCKMT	-4.83	N/A	N/A	N/A	N/A			✓	✓	✓
TGGT1_284620	hypothetical protein	-1.02	N/A	N/A	N/A	N/A	Conoid canopy ring	25	✓	✓	✓
TGGT1_212780	hypothetical protein	-5.33	N/A	N/A	N/A	N/A			✓	✓	✓
TGGT1_210430	DnaJ domain-containing protein	-3.81	N/A	N/A	N/A	N/A			✓	✓	✓
TGGT1_299190	B-box zinc finger domain-containing protein	-2.58	N/A	N/A	N/A	N/A			×	✓	×
TGGT1_310070	Apical annuli methyltransferase (AAMT)	-1.22	8.99E-20	cytosol	PM - peripheral 2	1	Apical annuli	36	✓	✓	✓
TGGT1_216080	apical complex lysine methyltransferase (AKMT)	-4.3	7.58E-21	cytosol	nucleolus	0.992	Conoid+preconidal rings	5, 13	✓	✓	✓
TGGT1_238170	hypothetical protein	-5.06	N/A	N/A	N/A	N/A			✓	✓	✓
TGGT1_293480	MoeA N-terminal region (domain I and II) domain-containing protein	-1.55	0.00164	nucleus - chromatin	nucleus - non-chromatin	0.827			×	✓	✓
TGGT1_253440	putative cell-cycle-associated protein kinase SRPK	-3.09	0.971	nucleus - chromatin	nucleus - chromatin	0.987			✓	✓	✓
TGGT1_202120	ICAP16	-2.1	0	nucleus - chromatin	PM - peripheral 2	0.985	Conoid canopy ring	25, 37	✓	✓	✓
TGGT1_230940	hypothetical protein	-4.92	0.667	nucleus - chromatin	nucleus - chromatin	0.928			×	✓	×
TGGT1_263070	CMGC kinase, CK2 family	-2.65	0.879	cytosol	cytosol	0.558			✓	×	✓
TGGT1_231160	hypothetical protein	-1.6	7.05E-19	IMC	IMC	0.698			✓	✓	✓
TGGT1_312630	GAC	-3.53	0.999	cytosol	cytosol	1	Conoid + cytosol	5, 18	×	✓	✓

Table S2: gRNAs used this study.

**gRNA name**	**Target Gene**	**gRNA sequence (5´- 3´)**	**Usage**	**Reference**
cgp-sgRNA1	*Hypothetical protein TGGT1_240380*	GAGCGGTGGAGGGTGGATTTC	tagging	24
cgp-sgRNA2	*Hypothetical protein TGGT1_240380*	GTGTCGACGCAGAGAAGTGGA	introduce 5' loxp	24
rng2-sgRNA	*rng2 (TGGT1_244470)*	GCATCAACAAACTAAAAAAA	tagging	this study
gac-sgRNA	*gac (TGGT1_312630)*	GTAAGGGCAGTCTCTGGAGTC	tagging	this study
frm1-sgRNA	*frm1 (TGGT1_462965)*	GAAATGAGTCTGAGACTTTC	tagging	this study
frm1-sgRNA	*frm1 (TGGT1_462965)*	GAAAGCGTATCAGACATGGT	Introduce 5' loxP	this study
myoh -sgRNA	*myoh (TGGT1_243250)*	GTCCCAGTTCGGATTCACCGA	tagging	this study
asaf1-sgRNA	*asaf1 (TGGT1_212780)*	gCGGGGAATGAAAGCCCCACA	tagging	this study
aamt-sgRNA	*aamt (TGGT1_310070)*	g CGACGAACTGAACCGGTGTG	tagging	this study
TGGT1_263070-sgRNA	*CMGC kinase, CK2 family (TGGT1_263070)*	gAAGTCCATGAAAAAGGTCTC	tagging	this study
dap1-sgRNA	*dap1 (TGGT1_284620)*	GAGATGCCTTAACTGTCGAA	tagging	this study
icap16-sgRNA	*icap16 (TGGT1_202120)*	GACATTGAATAAAACAGTGG	tagging	this study
TGGT1_231160-sgRNA	*hypothetical protein (TGGT1_231160)*	g ATTCTGCTGCTAAgtgcaaa	tagging	this study
TGGT1_238170-sgRNA	*hypothetical protein (TGGT1_238170)*	g TAAgcgaagagtcgaacctc	tagging	this study
TGGT1_253440-sgRNA	*putative cell-cycle-associated protein kinase SRPK (TGGT1_253440)*	gcatacacgtgTCACTGTTGC	tagging	this study
TGGT1_293480-sgRNA	*MoeA N-terminal region (domain I and II) domain-containing protein (TGGT1_293480)*	g TGAtctgcctcgcgagctct	tagging	this study
akmt-sgRNA	*akmt (TGGT1_216080)*	GGCCAGTTGAggaacaattg	tagging	this study
dap1 -sgRNA	*dap1 (TGGT1_284620)*	gTTCTTTGGTGGACGTGGAGA	Introduce 5' loxP	this study
akmt-sgRNA	*akmt (TGGT1_216080)*	gtgatgttgatggagagtcg	Introduce 5' loxP	this study
pckmt-sgRNA	*pckmt (TGGT1_292170)*	GAAGTAGcaaggatcaaagc	tagging	this study
pcr4-sgRNA	*pcr4 (TGGT1_201220)*	gCTTCTCGCAGGCTAGgaggc	tagging	this study
pcr4-sgRNA	*pcr4 (TGGT1_201220)*	gTCTCTGGCCCGCTGCATGTC	Introduce 5' loxP	this study
asaf1-sgRNA	*asaf1 (TGGT1_212780)*	GGTGCACCAGTGCCTTACTT	Introduce 5' loxP	this study
sfa2-sgRNA	*sfa2 (TGGT1_205670)*	gAGTCGACTAAAGAGTTGCCC	tagging	this study
TGGT1_210430-sgRNA	*DnaJ domain-containing protein (TGGT1_210430)*	gATTCTAACGAAGAAACACCG	tagging	this study
TGGT1_210430-sgRNA	*DnaJ domain-containing protein(TGGT1_210430)*	GAATAGTTTCTCGACGAGTG	Introduce 5' loxP	this study
TGGT1_238170-sgRNA	*iap3 (TGGT1_238170)*	gTAAgcgaagagtcgaacctc	tagging	this study
imc1-sgRNA	*IMC1(TGGT1_231640)*	GTACAAGTATACCACGCTGA	tagging	this study
Nuf2-sgRNA	*Nuf2 (TGME49_309380)*	GCCGAGTAGAGCACCGATCT	tagging	49
Chromo1-sgRNA	*Chromo1 (TGME49_068280)*	gCGACAGTGGACGAACCGGTC	tagging	49
tlap1-sgRNA	*tlap1 (TGGT1_286600)*	GCAAGCTGGCCAGTCCGAGG	tagging	this study
Nd6-sgRNA	*Nd6 (TGGT1_248640)*	gAGTGGGGGGGCAGGAGGCCT	tagging	this study
λ-tubulin-sgRNA	*γ-tubulin(TGGT1_226870)*	gTTGGCCACCCTGCATAATCG	tagging	this study

Table S3. Oligonculeotides used in this tudy

Oligo name	Sequence 5' - 3'	Purpose
pU6-gRNA-sequencing-fw	CCTTCGAACTCTCGAATGTC	Sequencing primers
cgp 3' tagging homology primer fwd	AAGAGGATGACCTAGCATGGAGACTAGCGGTGGAGGGTGGtTTcCTcGCTGCTAAAATTGGAAGTGGAGG	PCR amplification of tags for C-terminal tagging
cgp 3' tagging homology primer rvs	CGCCAGCCTGTCTATTATGAAAATGCAAAGTGTCTGATGAGGAAACCAGTATAACTTCGTATAATGTATGCTATACG	PCR amplification of tags for C-terminal tagging
cgp 3' tagging analytical primer fwd	TCGCTTCAGAAGTTTGGTGG	genotyping primers for C-terminal tagging
cgp 3' tagging analytical primer rvs	CATACAGTGGTTGTGGCACT	genotyping primers for C-terminal tagging
cgp 5' loxp insertion homology	TTTCTGAAGGACCTCTTGTCGACGCAGAGAAGTATAACTTCGTATAGCATACATTATACGAAGTTATGGAAGGAAGATG	repair templates for insertion of 5' loxp
cgp 5' loxp insertion analytical primer fwd	GCATACATTATACGAAGTTATGGAAGG	integration primers for insertion of 5' loxp
cgp 5' loxp insertion analytical primer rvs	CTCTCCTGTTCTTCTGTGGT	integration/genotyping primers for insertion of 5' loxp
cgp 5' loxp insertion analytical primer fwd	ATCTCTCTCGCCACTTTGAC	genotyping primers for insertion of 5' loxp
rng2 tagging homology primer fwd	CAGCGTGTTGTCTCGGACGCATCAACAAACGCTAAAATTGGAAGTGGAGG	PCR amplification of tags for C-terminal tagging
rng2 3' tagging homology primer rvs	cggccggtcattttaagttccacgatccctATAACTTCGTATAATGTATGCTATACG	PCR amplification of tags for C-terminal tagging
sfa2 3' tagging gRNA fwd	AAGTT gAGTCGACTAAAGAGTTGCCC G	gRNA for C-terminal tagging
sfa2 3' tagging gRNA rvs	AAAAC GGGCAACTCTTTAGTCGACTc A	gRNA for C-terminal tagging
sfa2 3' tagging homology primer fwd	ACCAGTACACGACAGCCTTACAGAAGGGCCTTCGAAATATAACAAGTCGCGCTAAAATTGGAAGTGGAGG	PCR amplification of tags for C-terminal tagging
sfa2 3' tagging homology primer rvs	TACAGAAAACAGAAAGATAGCGGAGTGAGAGAAGAGTCGACTAAAGAGTTATAACTTCGTATAATGTATGCTATACG	PCR amplification of tags for C-terminal tagging
sfa2 3' tagging analytical primer fwd	gtgtattcctggtggtcaaagc	genotyping primers for C-terminal tagging
sfa2 3' tagging analytical primer rvs	CGTGCAGTACGACAACATCTG	genotyping primers for C-terminal tagging
TGGT1_210430 3' tagging gRNA fwd	AAGTT gATTCTAACGAAGAAACACCG G	gRNA for C-terminal tagging
TGGT1_210430 3' tagging gRNA rvs	AAAAC CGGTGTTTCTTCGTTAGAATc A	gRNA for C-terminal tagging
TGGT1_210430 3' tagging homology primer fwd	CTCCGCGCGAGAAGGGAGGCGACCGGGTTTCTGCGGAGAACTGCGAATTC GCTAAAATTGGAAGTGGAGG	PCR amplification of tags for C-terminal tagging
TGGT1_210430 3' tagging homology primer rvs	CTGTCTCTCTAGCGACTTCTGGACTGCACTTTCGACTTTCCCTGCCTCGG ATAACTTCGTATAATGTATGCTATACG	PCR amplification of tags for C-terminal tagging
TGGT1_210430 3' tagging analytical primer fwd	GGAAACCGGAAGAGAGAAGG	genotyping primers for C-terminal tagging
TGGT1_210430 3' tagging analytical primer rvs	CTCTTCCGTCTGTCTCTGTG	genotyping primers for C-terminal tagging
TGGT1_210430 5' upstream loxP gRNA fwd	AAGTT GAATAGTTTCTCGACGAGTG G	gRNA for 5' loxP insertion
TGGT1_210430 5' upstream loxP gRNA rvs	AAAAC CACTCGTCGAGAAACTATTC A	gRNA for 5' loxP insertion
TGGT1_210430 5' loxp insertion homology	CCTACACTGTAAAATTGCTTGTCTGCGCCTCACATAACTTCGTATAGCATACATTATACGAAGTTATTCGTCGAGAAACT	repair templates for insertion of 5' loxp
TGGT1_210430 5' loxp insertion analytical primer fwd	GCATACATTATACGAAGTTATTCGTCG	integration primers for insertion of 5' loxp
TGGT1_210430 5' loxp insertion analytical primer rvs	TTTCGAAGAAGACTTAGAGAGC	integration/genotyping primers for insertion of 5' loxp
TGGT1_210430 5' loxp insertion analytical primer fwd	TTCTTCAAACATGTTGTCTGGA	genotyping primers for insertion of 5' loxp
imc1 3' tagging gRNA fwd	AAGTTGTACAAGTATACCACGCTGAG	gRNA for C-terminal tagging
imc1 3' tagging gRNA rvs	AAAACTCAGCGTGGTATACTTGTACA	gRNA for C-terminal tagging
imc1 3' tagging homology primer fwd	GCGTCATGTGCGCGGTCTGCGGCGGTGATGGTGTGTGCCGATGCCAGTGC GCTAAAATTGGAAGTGGAGG	PCR amplification of tags for C-terminal tagging
imc1 3' tagging homology primer rvs	GAGGTCAGATTCCAGAAATTCCCGTCGCACAAGGCCTTCAGCGTGGTATA ATAACTTCGTATAATGTATGCTATACG	PCR amplification of tags for C-terminal tagging
imc1 3' tagging analytical primer fwd	GATCGAAGAGGCACATGCTG	genotyping primers for C-terminal tagging
imc1 3' tagging analytical primer rvs	ACTTGAAATTCCCGTGCCAG	genotyping primers for C-terminal tagging
pcr4 5' upstream loxP gRNA fwd	AAGTT gTCTCTGGCCCGCTGCATGTC G	gRNA for 5' loxP insertion
pcr4 5' upstream loxP gRNA rvs	AAAAC GACATGCAGCGGGCCAGAGAc A	gRNA for 5' loxP insertion
pcr4 5' loxp insertion homology	TCTTCTCTCTCCTCTCTCTCTGGCCCGCTGCAT ATAACTTCGTATAGCATACATTATACGAAGTTAT c GTCGGGGACGC	repair templates for insertion of 5' loxp
pcr4 5' loxp insertion analytical primer fwd	GCATACATTATACGAAGTTATcGTCGGG	integration primers for insertion of 5' loxp
pcr4 5' loxp insertion analytical primer rvs	CACACTCATTTGCAGGAGGTACTC	integration/genotyping primers for insertion of 5' loxp
pcr4 5' loxp insertion analytical primer fwd	GTTCTCTGGTCCCTCTCTTCCTC	genotyping primers for insertion of 5' loxp
MyoH 3' tagging homology primer fwd	CGCGAGGCGCTGCTGCTGCTCCCTCTGCAATCCGCACCCGTCCGTCGGTaAACCCaAAtTGGGAcCCgATGGCgTAtAAt G	PCR amplification of tags for C-terminal tagging
MyoH 3' tagging homology primer rvs	AAGAAAGGCCTTTTTTCAACACTCACCAGCGGGCAGCCGCCAAAAAGATC ATAACTTCGTATAATGTATGCTATACG	PCR amplification of tags for C-terminal tagging
MyoH 3' tagging analytical primer fwd	CGCCGTGTACCAATACATTC	genotyping primers for C-terminal tagging
MyoH 3' tagging analytical primer rvs	GAGAACAACTGACGCAAGAC	genotyping primers for C-terminal tagging
cgp 3' tagging gRNA fwd	AAGTTgAGCGGTGGAGGGTGGATTTCG	gRNA for C-terminal tagging
cgp 3' tagging gRNA rvs	AAAACGAAATCCACCCTCCACCGCTcA	gRNA for C-terminal tagging
cgp 5' upstream loxP gRNA fwd	AAGTT gTGTCGACGCAGAGAAGTGGA G	gRNA for 5' loxP insertion
cgp 5' upstream loxP gRNA rvs	AAAAC TCCACTTCTCTGCGTCGACAc A	gRNA for 5' loxP insertion
rng2 3' tagging gRNA fwd	AAGTT GCATCAACAAACTAAAAAAA G	gRNA for C-terminal tagging
rng2 3' tagging gRNA rvs	AAAAC TTTTTTTAGTTTGTTGATGC A	gRNA for C-terminal tagging
frm1 3' tagging gRNA fwd	AAGTTGAAATGAGTCTGAGACTTTCG	gRNA for C-terminal tagging
frm1 3' tagging gRNA rvs	AAAACGAAAGTCTCAGACTCATTTCA	gRNA for C-terminal tagging
frm1 3' tagging homology primer fwd	caactcccgtggcgctgcttgtgtgtgtgttcaataacagGGATGAAAGCTAAAATTGGAAGTGGAGGA	PCR amplification of tags for C-terminal tagging
frm1 3' tagging homology primer rvs	GTCGACCAACAGTCGCTCCTTCAGCACCTGACCACTGAGCGCTTCCGGAAATAACTTCGTATAATGTATGCTATACG	PCR amplification of tags for C-terminal tagging
frm1 3' tagging analytical primer fwd	GACTCAAGTCGACTGCAGTC	genotyping primers for C-terminal tagging
frm1 3' tagging analytical primer rvs	TGGCATTGCCTAAATGATTG	genotyping primers for C-terminal tagging
frm1 5' upstream loxP gRNA fwd	AAGTTGAAAGCGTATCAGACATGGTG	gRNA for 5' loxP insertion
frm1 5' upstream loxP gRNA rvs	AAAACACCATGTCTGATACGCTTTCA	gRNA for 5' loxP insertion
frm1 5' loxp insertion homology	CCCAAGCACCGATTTGGAGTCTCTAGTCCCACCATAACTTCGTATAGCATACATTATACGAAGTTATATGTCTGATACG	repair templates for insertion of 5' loxp
frm1 5' loxp insertion analytical primer fwd	TCTAGTCCCACCATAACTTCG	integration primers for insertion of 5' loxp
frm1 5' loxp insertion analytical primer rvs	AGACTACCAGCACGTGTG	integration/genotyping primers for insertion of 5' loxp
frm1 5' loxp insertion analytical primer fwd	CTCTGTCTGGGAAGTTTCTTACC	genotyping primers for insertion of 5' loxp
gac 3' tagging gRNA fwd	AAGTTGTAAGGGCAGTCTCTGGAGTCG	gRNA for C-terminal tagging
gac 3' tagging gRNA rvs	AAAACGACTCCAGAGACTGCCCTTACA	gRNA for C-terminal tagging
gac 3' tagging homology primer fwd	CATTTGTCGAGATGATGGTGCAGTGGCGTGACGCTGCAACGTACAATTTTGCTAAAATTGGAAGTGGAGG	PCR amplification of tags for C-terminal tagging
gac 3' tagging homology primer rvs	CGGCGTTGACTAGCGTCCGGTCGCGTCCTCAGGCGCAGCGGCCGCCGGACATAACTTCGTATAATGTATGCTATACG	PCR amplification of tags for C-terminal tagging
gac 3' tagging analytical primer fwd	CATGGTCGCGAAGAGTCAG	genotyping primers for C-terminal tagging
gac 3' tagging analytical primer rvs	ACTTGCCGAACTCCAAGG	genotyping primers for C-terminal tagging
myoh 3' tagging gRNA fwd	AAGTTgTCCCAGTTCGGATTCACCGAG	gRNA for C-terminal tagging
myoh 3' tagging gRNA rvs	AAAACTCGGTGAATCCGAACTGGGAcA	gRNA for C-terminal tagging
myoh 3' tagging homology primer fwd	CGCGAGGCGCTGCTGCTGCTCCCTCTGCAATCCGCACCCGTCCGTCGGTaAACCCaAAtTGGGAcCCgATGGCgTAtAAt G	PCR amplification of tags for C-terminal tagging
myoh 3' tagging homology primer rvs	AAGAAAGGCCTTTTTTCAACACTCACCAGCGGGCAGCCGCCAAAAAGATC ATAACTTCGTATAATGTATGCTATACG	PCR amplification of tags for C-terminal tagging
myoh 3' tagging analytical primer fwd	CGCCGTGTACCAATACATTC	genotyping primers for C-terminal tagging
myoh 3' tagging analytical primer rvs	GAGAACAACTGACGCAAGAC	genotyping primers for C-terminal tagging
pcr4 3' tagging gRNA fwd	AAGTT g CTTCTCGCAGGCTAGgaggc G	gRNA for C-terminal tagging
pcr4 3' tagging gRNA rvs	AAAAC gcctcCTAGCCTGCGAGAAG c A	gRNA for C-terminal tagging
pcr4 3' tagging homology primer fwd	GGAAGAGTCCGGAAAAGAGAGTCCACTTCGGCTCCACACTTCTCGCAGGCGCTAAAATTGGAAGTGGAGG	PCR amplification of tags for C-terminal tagging
pcr4 3' tagging homology primer rvs	tgggatcgtcctcttcgttccgtctcggcgcgcctctggatgtccccgcc ATAACTTCGTATAATGTATGCTATACG	PCR amplification of tags for C-terminal tagging
pcr4 3' tagging analytical primer fwd	tgctctgcaggccgtttctgtg	genotyping primers for C-terminal tagging
pcr4 3' tagging analytical primer rvs	ctgccattcgagcgttcgtcgt	genotyping primers for C-terminal tagging
TGGT1_212780 3' tagging gRNA fwd	AAGTT gCGGGGAATGAAAGCCCCACA G	gRNA for C-terminal tagging
TGGT1_212780 3' tagging gRNA rvs	AAAAC TGTGGGGCTTTCATTCCCCGc A	gRNA for C-terminal tagging
TGGT1_212780 3' tagging homology primer fwd	AGAAAGTACAGGAACTCCGGAGACAACTGAGGGAACATGGGGTGCTATCTGCTAAAATTGGAAGTGGAGG	PCR amplification of tags for C-terminal tagging
TGGT1_212780 3' tagging homology primer rvs	CAGGAACTGTGGCCTGTGAATCTGCCGTCTGTGCGGGGAATGAAAGCCCCATAACTTCGTATAATGTATGCTATACG	PCR amplification of tags for C-terminal tagging
TGGT1_212780 3' tagging analytical primer fwd	CAGTTCAGGTGACTTTGTGCAG	genotyping primers for C-terminal tagging
TGGT1_212780 3' tagging analytical primer rvs	CGTTTCACTTGCGATTCTGG	genotyping primers for C-terminal tagging
aamt 3' tagging gRNA fwd	AAGTT g CGACGAACTGAACCGGTGTG G	gRNA for C-terminal tagging
aamt 3' tagging gRNA rvs	AAAAC CACACCGGTTCAGTTCGTCGc A	gRNA for C-terminal tagging
aamt 3' tagging homology primer fwd	AAGCCCCTGCACAGGTGTCCTCGGATGCCGCCAAAGGTGCCCCGACGAACGCTAAAATTGGAAGTGGAGG	PCR amplification of tags for C-terminal tagging
aamt 3' tagging homology primer rvs	TATGTGTTGCTTTCGGCTTATATGCGGCCCACTTTCCCCAGTCTCCACACATAACTTCGTATAATGTATGCTATACG	PCR amplification of tags for C-terminal tagging
aamt 3' tagging analytical primer fwd	cacatcgtgtgggtagatcg	genotyping primers for C-terminal tagging
aamt 3' tagging analytical primer rvs	GCAGAACAGAGTCGAAGTCTAC	genotyping primers for C-terminal tagging
TGGT1_263070 3' tagging gRNA fwd	AAGTT gAAGTCCATGAAAAAGGTCTC G	gRNA for C-terminal tagging
TGGT1_263070 3' tagging gRNA rvs	AAAAC GAGACCTTTTTCATGGACTTc A	gRNA for C-terminal tagging
TGGT1_263070 3' tagging homology primer fwd	AAGAAGAGCAACAGAAGGCAGCGAACCTTTCGGCGTCGAGCGTGAAAGCCGCTAAAATTGGAAGTGGAGG	PCR amplification of tags for C-terminal tagging
TGGT1_263070 3' tagging homology primer rvs	AGCAAAAAGTCCCCTCCGGTCGATCATAAAAAAAAGTCCATGAAAAAGGT ATAACTTCGTATAATGTATGCTATACG	PCR amplification of tags for C-terminal tagging
TGGT1_263070 3' tagging analytical primer fwd	tttcagtgttgagtggcgac	genotyping primers for C-terminal tagging
TGGT1_263070 3' tagging analytical primer rvs	CACCTACAGCATCTCTGCACTC	genotyping primers for C-terminal tagging
dap1 3' tagging gRNA fwd	AAGTT GAGATGCCTTAACTGTCGAA G	gRNA for C-terminal tagging
dap1 3' tagging gRNA rvs	AAAAC TTCGACAGTTAAGGCATCTC A	gRNA for C-terminal tagging
dap1 3' tagging homology primer fwd	GTGAAGGCTCTAAAAACAATAGCGATGCTGAAAGGTCCGAAAAGAGCGAC GCTAAAATTGGAAGTGGAGG	PCR amplification of tags for C-terminal tagging

dap1 3' tagging homology primer rvs	AAGAGATACCAGCTGCATGCACAAAAATAGATGCTTCATCCTCACCATTC ATAACTTCGTATAATGTATGCTATACG	PCR amplification of tags for C-terminal tagging
dap1 3' tagging analytical primer fwd	CAAAGCAGAACAGGATGCAGG	genotyping primers for C-terminal tagging
dap1 3' tagging analytical primer rvs	GATATAAGCACAACTCTCTCGCG	genotyping primers for C-terminal tagging
icap16 3' tagging gRNA fwd	AAGTT GACATTGAATAAAACAGTGG G	gRNA for C-terminal tagging
icap16 3' tagging gRNA rvs	AAAAC CCACTGTTTTATTCAATGTC A	gRNA for C-terminal tagging
icap16 3' tagging homology primer fwd	AAGATGACGCTCAGGATCTTTCTTCGCGGTCCTCTACCAGCGGCAGCGGT GCTAAAATTGGAAGTGGAGG	PCR amplification of tags for C-terminal tagging
icap16 3' tagging homology primer rvs	GCGCACTTCTCGCCGAAGGCAGTCAGTCCAATCTTTGATTTACTCCTCCA ATAACTTCGTATAATGTATGCTATACG	PCR amplification of tags for C-terminal tagging
icap16 3' tagging analytical primer fwd	GGACGGCGTGTACTTTAATC	genotyping primers for C-terminal tagging
icap16 3' tagging analytical primer rvs	CTCATTCTGTAAGCCGTTGC	genotyping primers for C-terminal tagging
TGGT1_231160 3' tagging gRNA fwd	AAGTTg ATTCTGCTGCTAAgtgcaaa G	gRNA for C-terminal tagging
TGGT1_231160 3' tagging gRNA rvs	AAAAC tttgcacTTAGCAGCAGAAT c A	gRNA for C-terminal tagging
TGGT1_231160 3' tagging homology primer fwd	CGGCTGCCAAGAAGATCTCCAAGAGCTCCAAGTCACGGGGATTCTGCTGCGCTAAAATTGGAAGTGGAGG	PCR amplification of tags for C-terminal tagging
TGGT1_231160 3' tagging homology primer rvs	agcacgtcgaaggaactgggaagagcagcggcgtcgcacgcatgcccttt ATAACTTCGTATAATGTATGCTATACG	PCR amplification of tags for C-terminal tagging
TGGT1_231160 3' tagging analytical primer fwd	cgtgcagagagtgggggagaca	genotyping primers for C-terminal tagging
TGGT1_231160 3' tagging analytical primer rvs	agcagccagagcgagggagaaa	genotyping primers for C-terminal tagging
TGGT1_238170 3' tagging gRNA fwd	AAGTT g TAAgcgaagagtcgaacctc G	gRNA for C-terminal tagging
TGGT1_238170 3' tagging gRNA rvs	AAAAC gaggttcgactcttcgcTTA c A	gRNA for C-terminal tagging
TGGT1_238170 3' tagging homology primer fwd	TCGCCATCGCCACGAAATCGCGTCTCTTCTCGCTCTTACACGACCTTCTT GCTAAAATTGGAAGTGGAGG	PCR amplification of tags for C-terminal tagging
TGGT1_238170 3' tagging homology primer rvs	gtatacgtatgagaacgtatttgagtgtttgtaagcatgtttatcccgagATAACTTCGTATAATGTATGCTATACG	PCR amplification of tags for C-terminal tagging
TGGT1_238170 3' tagging analytical primer fwd	TCTCCTCTCGCTCGTCAACGCA	genotyping primers for C-terminal tagging
TGGT1_238170 3' tagging analytical primer rvs	cgtagactgctgccagctctgc	genotyping primers for C-terminal tagging
TGGT1_253440 3' tagging gRNA fwd	AAGTT gcatacacgtgTCACTGTTGC G	gRNA for C-terminal tagging
TGGT1_253440 3' tagging gRNA rvs	AAAAC GCAACAGTGAcacgtgtatg c A	gRNA for C-terminal tagging
TGGT1_253440 3' tagging homology primer fwd	TCGGAAAGGCACAACAACAATTTCAACAACAGCAACAGCTCCAGCAACAG GCTAAAATTGGAAGTGGAGG	PCR amplification of tags for C-terminal tagging
TGGT1_253440 3' tagging homology primer rvs	gctttgccgcatcacgcaaactgtgcattcccagcgcacgcatacacgtgATAACTTCGTATAATGTATGCTATACG	PCR amplification of tags for C-terminal tagging
TGGT1_253440 3' tagging analytical primer fwd	TACCAGGCGCGTCACCAACAAC	genotyping primers for C-terminal tagging
TGGT1_253440 3' tagging analytical primer rvs	tgcgcgaaaaagggagatgcgt	genotyping primers for C-terminal tagging
TGGT1_293480 3' tagging gRNA fwd	AAGTT g TGAtctgcctcgcgagctct G	gRNA for C-terminal tagging
TGGT1_293480 3' tagging gRNA rvs	AAAACagagctcgcgaggcagaTCA c A	gRNA for C-terminal tagging
TGGT1_293480 3' tagging homology primer fwd	CCCTGTTGCCGGTTTTACCCCACGCGTTGGAAGTTGTCACTTCGGGGGCGGCTAAAATTGGAAGTGGAGG	PCR amplification of tags for C-terminal tagging
TGGT1_293480 3' tagging homology primer rvs	ggaacggacactgcggcatatatttagccatcaagtagaagtcgcctaga ATAACTTCGTATAATGTATGCTATACG	PCR amplification of tags for C-terminal tagging
TGGT1_293480 3' tagging analytical primer fwd	TTCGGTGCGAGACGTCACTCCT	genotyping primers for C-terminal tagging
TGGT1_293480 3' tagging analytical primer rvs	ggcgactgacttccccaaacgg	genotyping primers for C-terminal tagging
akmt 3' tagging gRNA fwd	AAGTT GGCCAGTTGAggaacaattg G	gRNA for C-terminal tagging
akmt 3' tagging gRNA rvs	AAAAC caattgttccTCAACTGGCC A	gRNA for C-terminal tagging
akmt 3' tagging homology primer fwd	TCGCTGCCGGAACCAAAAAAACTGCATGCGAGGAACGCCTACCGGCCAGT GCTAAAATTGGAAGTGGAGG	PCR amplification of tags for C-terminal tagging
akmt 3' tagging homology primer rvs	agccctttaccccttgcgaacacgagaagtcctccgccagtctcccacaa ATAACTTCGTATAATGTATGCTATACG	PCR amplification of tags for C-terminal tagging
akmt 3' tagging analytical primer fwd	CGGCCCACAAGCTGAACGTGAT	genotyping primers for C-terminal tagging
akmt 3' tagging analytical primer rvs	tttcgacagagtgcacacggcc	genotyping primers for C-terminal tagging
akmt 5' upstream loxP gRNA fwd	AAGTT gtgatgttgatggagagtcg G	gRNA for 5' loxP insertion
akmt 5' upstream loxP gRNA rvs	AAAAC cgactctccatcaacatcac A	gRNA for 5' loxP insertion
akmt 5' loxp insertion homology	gagccgtgcccctggtgaccacagtaccctcgaATAACTTCGTATAGCATACATTATACGAAGTTATctctccatcaacatcacgtcttaatctaacaga	repair templates for insertion of 5' loxp
akmt 5' loxp insertion analytical primer fwd	GCATACATTATACGAAGTTATctctccatc	integration primers for insertion of 5' loxp
akmt 5' loxp insertion analytical primer rvs	TTCCCCGTAGTCGTCACCGCTT	integration/genotyping primers for insertion of 5' loxp
akmt 5' loxp insertion analytical primer fwd	acgtgtggaatgccttgcctcg	genotyping primers for insertion of 5' loxp
dap1 5' upstream loxP gRNA fwd	AAGTT gTTCTTTGGTGGACGTGGAGA G	gRNA for 5' loxP insertion
dap1 5' upstream loxP gRNA rvs	AAAAC TCTCCACGTCCACCAAAGAAc A	gRNA for 5' loxP insertion
dap1 5' loxp insertion homology	CCGAATCTTCAGGCTTTTTCGATTCTTCCCTCTATAACTTCGTATAGCATACATTATACGAAGTTATCCACGTCCACCAA[illegible]	repair templates for insertion of 5' loxp
dap1 5' loxp insertion analytical primer fwd	GCATACATTATACGAAGTTATCCACG	integration primers for insertion of 5' loxp
dap1 5' loxp insertion analytical primer rvs	CTCAACATCGACATCCAAATCACC	integration/genotyping primers for insertion of 5' loxp
dap1 5' loxp insertion analytical primer fwd	CTCTAATTGCCTCACCAGTC	genotyping primers for insertion of 5' loxp
pckmt 3' tagging gRNA fwd	AAGTTGAAGTAGcaaggatcaaagcG	gRNA for C-terminal tagging
pckmt 3' tagging gRNA rvs	AAAACcccgctttgatccttgCTACTTCA	gRNA for C-terminal tagging
pckmt 3' tagging homology primer fwd	AGAAAGGTCCGCCTTTGCTTGGGAAGAAAGGTTTACCACCTCCAAAGAAGGCTAAAATTGGAAGTGGAGG	PCR amplification of tags for C-terminal tagging
pckmt 3' tagging homology primer rvs	caaaactgtcgaaaaccgggccatgaaatgcgagtgccccttttgcccgctATAACTTCGTATAATGTATGCTATACG	PCR amplification of tags for C-terminal tagging
pckmt 3' tagging analytical primer fwd	AAGAAAGCTCCACCGCCTCCAC	genotyping primers for C-terminal tagging
pckmt 3' tagging analytical primer rvs	agcaaacgcggaattctgggca	genotyping primers for C-terminal tagging
TGGT1_212780 5' upstream loxP gRNA fwd	AAGTT GGTGCACCAGTGCCTTACTT G	gRNA for 5' loxP insertion
TGGT1_212780 5' upstream loxP gRNA rvs	AAAAC AAGTAAGGCACTGGTGCACC A	gRNA for 5' loxP insertion
TGGT1_212780 5' loxp insertion homology	GTTCTCTCCAGATTTAGGTGCACCAGTGCCTTAATAACTTCGTATAGCATACATTATACGAAGTTATCTTGGGACCTCTT	repair templates for insertion of 5' loxp
TGGT1_212780 5' loxp insertion analytical primer fwd	GCATACATTATACGAAGTTATCTTGGG	integration primers for insertion of 5' loxp
TGGT1_212780 5' loxp insertion analytical primer rvs	ATCTGTTGGACTCCTGGTTATA	integration/genotyping primers for insertion of 5' loxp
TGGT1_212780 5' loxp insertion analytical primer fwd	CTGTCCTTGTTCGAGTCTTTAT	genotyping primers for insertion of 5' loxp
nuf2 3' tagging gRNA fwd	AAGTTGCCGAGTAGAGCACCGATCTG	gRNA for C-terminal tagging
nuf2 3' tagging gRNA rvs	AAAACAGATCGGTGCTCTACTCGGCA	gRNA for C-terminal tagging
nuf2 3' tagging homology primer fwd	CAAGGGAGCCCAGAGAAGACGGCGACTTTCCAATGTATAGTCACGCCGAGGCTAAAATTGGAAGTGGAGG	PCR amplification of tags for C-terminal tagging
nuf2 3' tagging homology primer rvs	GCCAAAGTTCTCCGAGTGTCCGTACACCGGAAACTTTCTCCATGCCAAGAATAACTTCGTATAATGTATGCTATACG	PCR amplification of tags for C-terminal tagging
nuf2 3' tagging analytical primer fwd	AGCGAGAACGAGAATCCGAC	genotyping primers for C-terminal tagging
nuf2 3' tagging analytical primer rvs	CGAGTGTCCGTACACCGGAA	genotyping primers for C-terminal tagging
chromo1 3' tagging gRNA fwd	AAGTT gCGACAGTGGACGAACCGGTC G	gRNA for C-terminal tagging
chromo1 3' tagging gRNA rvs	AAAAC GACCGGTTCGTCCACTGTCGc A	gRNA for C-terminal tagging
chromo1 3' tagging homology primer fwd	CCGTCTGCGGGAGGTTCCAACGCTCTCCAGTGTTCTCTTGTGTCGCTGGC GCTAAAATTGGAAGTGGAGG	PCR amplification of tags for C-terminal tagging
chromo1 3' tagging homology primer rvs	CCAGTCTCGTGGAAGCGGTGCAACGCCTCTGGGATTCCGTTCGGCCTGAC ATAACTTCGTATAATGTATGCTATACG	PCR amplification of tags for C-terminal tagging
chromo1 3' tagging analytical primer fwd	CGTTCGTTTCAGATGAGTCC	genotyping primers for C-terminal tagging
chromo1 3' tagging analytical primer rvs	GGTATAGAGACAGCGCGTTG	genotyping primers for C-terminal tagging
Tlap1 3' tagging gRNA fwd	AAGTTGCAAGCTGGCCAGTCCGAGGG	gRNA for C-terminal tagging
Tlap1 3' tagging gRNA rvs	AAAACCCTCGGACTGGCCAGCTTGCA	gRNA for C-terminal tagging
Tlap1 3' tagging homology primer fwd	GTGACTTCCTCGAGTTgCGCgTCGGACTGGCCAGCTTGCTCTTTCTCGCA ATGGTGAGCAAGGGCGAG	PCR amplification of tags for C-terminal tagging
Tlap1 3' tagging homology primer rvs	TGTGCCGTGCCCGGGATTGGCACTGCAAGGGGTCCACCCACCGACGTCGCTCCGCCGCTGCCACCCCCTCCACTGCCA[illegible]	PCR amplification of tags for C-terminal tagging
Tlap1 3' tagging analytical primer fwd	TGTTCTGTGCTAGACAGGTG	genotyping primers for C-terminal tagging
Tlap1 3' tagging analytical primer rvs	gtacagacccacagatcacac	genotyping primers for C-terminal tagging
Nd6 3' tagging gRNA fwd	AAGTT gAGTGGGGGGGCAGGAGGCCT G	gRNA for C-terminal tagging
Nd6 3' tagging gRNA rvs	AAAAC AGGCCTCCTGCCCCCCCACTc A	gRNA for C-terminal tagging
Nd6 3' tagging homology primer fwd	ACGTTCTGCCCTTTCTCCAAGCGCTCGACCAAGTGGGGGGGCAGGAGGCC GCTAAAATTGGAAGTGGAGG	PCR amplification of tags for C-terminal tagging
Nd6 3' tagging homology primer rvs	GTTGGCGTGGCTGCGCCGGCTTTCTGTTTTATGCGCTCTACTGTGGAGGC ATAACTTCGTATAATGTATGCTATACG	PCR amplification of tags for C-terminal tagging
Nd6 3' tagging analytical primer fwd	GTAGCCGACTTTGTCATTCC	genotyping primers for C-terminal tagging
Nd6 3' tagging analytical primer rvs	CAAGAAACTACGCTGAGCAG	genotyping primers for C-terminal tagging
γ- tubulin 3' tagging gRNA fwd	AAGTT gTTGGCCACCCTGCATAATCG G	gRNA for C-terminal tagging
γ- tubulin 3' tagging gRNA rvs	AAAAC CGATTATGCAGGGTGGCCAAc A	gRNA for C-terminal tagging
γ- tubulin 3' tagging homology primer fwd	ggcttctctctcggcttcccagGTGACTTTGGCGTTGGCCACCCTGCA GCTAAAATTGGAAGTGGAGG	PCR amplification of tags for C-terminal tagging
γ- tubulin 3' tagging homology primer rvs	TTGAGTCTTCGTCGTTCGTCGTCATCGACGCAGTTCTTTGGCAGTTCTCC ATAACTTCGTATAATGTATGCTATACG	PCR amplification of tags for C-terminal tagging
γ- tubulin 3' tagging analytical primer fwd	cagcaccgagagtttcatattc	genotyping primers for C-terminal tagging
γ- tubulin 3' tagging analytical primer rvs	GCAGCTCCACAGTATAGTCA	genotyping primers for C-terminal tagging

Table S4. *T. gondii* strains generated/used in this study.

**Strains**	**Information**	**Application**	**Reference**
DiCreΔKu80	Expressing DiCre subunits. *Ku80* is KO	All strains in this study are DiCreΔKu80 background.WT control for TurboID	50
loxP*cgp* -Halo	*Cgp* is floxed and C-terminally tagged with Halo	Inducible KO of *cgp*	24
loxP*cgp* -Halo/*myoh* -SYFP2	*Cgp* is floxed and C-terminally tagged with Halo. *Myoh* is C-terminally tagged with SYFP2	Inducible KO of *cgp* and analysis of its effect on MyoH	this study
loxP*cgp* -Halo/*akmt* -SYFP2	*Cgp* is floxed and C-terminally tagged with Halo. *Akmt* is C-terminally tagged with SYFP2	Inducible KO of *cgp* and analysis of its effect on AKMT	this study
loxP*cgp* -Halo/*gac* -SYFP2	*Cgp* is floxed and C-terminally tagged with Halo. *Gac* is C-terminally tagged with SYFP2	Inducible KO of *cgp* and analysis of its effect on GAC	this study
loxP*cgp* -Halo/*frm1* -SNAP	*Cgp* is floxed and C-terminally tagged with Halo. *Frm1* is C-terminally tagged with SNAP	Inducible KO of *cgp* and analysis of its effect on FRM1	this study
loxP*frm1* -mCherry/*cgp* -Halo	*Frm1* is floxed and C-terminally tagged with mCherry. *Cgp* is C-terminally tagged with Halo	Inducible KO of *frm1* and analysis of its effect on CGP	this study
loxP*frm1* -Halo/gac-SYFP2	*Frm1* is floxed and C-terminally tagged with Halo. *Gac* is C-terminally tagged with SYFP2	Inducible KO of *frm1* and analysis of its effect on GAC	this study
loxP*cgp* -Halo/*pcr4* -SNAP	*Cgp* is floxed and C-terminally tagged with Halo. *Pcr4* is C-terminally tagged with SNAP	STED imaging anaysis pf CGP and PCR4	this study
loxP*cgp* -Halo/*pcr4* -SYFP2	*Cgp* is floxed and C-terminally tagged with Halo. *Pcr4* is C-terminally tagged with SYFP2	Inducible KO of *cgp* and analysis of its effect on PCR4	this study
loxP*pcr4* -SYFP2/*cgp* -Halo	*Pcr4* is floxed and C-terminally tagged with SYFP2. *Cgp* is C-terminally tagged with Halo	Inducible KO of *pcr4* and analysis of its effect on CGP	this study
loxP*cgp* -Halo/*frm1* -3HA	Cgp is floxed and C-terminally tagged with Halo. Frm1 is C-terminally tagged with 3HA	Inducible KO of *cgp* and analysis of its effect on FRM1	this study
loxP*cgp* -Halo/*pcr4* -Halo	*Cgp* is floxed and C-terminally tagged with Halo. *Pcr4* is C-terminally tagged with Halo	Inducible KO of *cgp* and analysis of its effect on apical structures by CryoET	this study
cgp-TurboID/frm1-3HA	*Cgp* is C-terminally tagged with TurboID. *Frm1* is C-terminally tagged with 3HA	CGP TurboID	this study
frm1 -TurboID/loxP*cgp* -Halo	*Frm1* is C-terminally tagged with TurboID. *Cgp* is floxed and C-terminally tagged with Halo	FRM1 TurboID	this study
253440-SYFP2	253440 is C-terminally tagged with SYFP2 in loxPcgp-Halo background	Analysis of protein localisation	this study
231160-SYFP2	231160 is C-terminally tagged with SYFP2 in loxPcgp-Halo background	Analysis of protein localisation	this study
263070-SYFP2	263070 is C-terminally tagged with SYFP2 in loxPcgp-Halo background	Analysis of protein localisation	this study
210430-3HA	210430 is C-terminally tagged with 3HA	Analysis of protein localisation	this study
212780-3HA	212780 is C-terminally tagged with 3HA in loxPcgp-Halo background	Analysis of protein localisation	this study
293480-SYFP2	293480 is C-terminally tagged with SYFP2 in loxPfrm1-Halo background	Analysis of protein localisation	this study
loxP*cgp* -Halo/aamt-SYFP2	*Cgp* is floxed and C-terminally tagged with Halo. *Aamt* is C-terminally tagged with SYFP2	Inducible KO of *cgp* and analysis of its effect on AAMT	this study
loxP*frm1* -Halo/aamt-SYFP2	*Frm1* is floxed and C-terminally tagged with Halo. *Aamt* is C-terminally tagged with SYFP2	Inducible KO of *frm1* and analysis of its effect on AAMT	this study
loxP*frm1* -Halo/*akmt* -SYFP2	*Frm1* is floxed and C-terminally tagged with Halo. *Akmt* is C-terminally tagged with SYFP2	Inducible KO of *frm1* and analysis of its effect on AkMT	this study
loxP*akmt* -SYFP2/*cgp* -Halo	*Akmt* is floxed and C-terminally tagged with SYFP2. *Cgp* is C-terminally tagged with Halo	Inducible KO of *akmt* and analysis of its effect on CGP	this study
loxP*akmt* -SYFP2/*frm1* -Halo	*Akmt is floxed and C-terminally tagged with SYFP2. Frm1* is C-terminally tagged with Halo	Inducible KO of *akmt* and analysis of its effect on FRM1	this study
loxP*frm1* -Halo/*icap16* -SYFP2	*Frm1* is floxed and C-terminally tagged with Halo. *Icap16* is C-terminally tagged with SYFP2	Inducible KO of *frm1* and analysis of its effect on ICAP16	this study
loxP*cgp* -Halo/*icap16* -SYFP2	*Cgp* is floxed and C-terminally tagged with Halo. *Icap16* is C-terminally tagged with SYFP2	Inducible KO of *cgp* and analysis of its effect on ICAP16	this study
loxP*cgp* -Halo/*dap1* -SYFP2	*Cgp* is floxed and C-terminally tagged with Halo. *Dap1* is C-terminally tagged with SYFP2	Inducible KO of *cgp* and analysis of its effect on Dap1	this study
loxP*frm1* -Halo/*dap1* -SYFP2	*Frm1* is floxed and C-terminally tagged with Halo. *Dap1* is C-terminally tagged with SYFP2	Inducible KO of *frm1* and analysis of its effect on Dap1	this study
loxP*dap1* -SYFP2/*cgp* -Halo	*Dap1* is floxed and C-terminally tagged with SYFP2. *Cgp* is C-terminally tagged with Halo	Inducible KO of *dap1* and analysis of its effect on CGP	this study
loxP*dap1* -SYFP2/*frm1* -Halo	*Dap1* is floxed and C-terminally tagged with SYFP2. *FRM1* is C-terminally tagged with Halo	Inducible KO of *dap1* and analysis of its effect on FRM1	this study
loxP*dap1* -SYFP2/*aamt* -Halo	*Dap1* is floxed and C-terminally tagged with SYFP2. *Aamt* is C-terminally tagged with Halo	Inducible KO of *dap1* and analysis of its effect on AAMT	this study
loxP*dap1* -SYFP2/*icap16* -Halo	*Dap1* is floxed and C-terminally tagged with SYFP2. *Icap16* is C-terminally tagged with Halo	Inducible KO of *dap1* and analysis of its effect on ICAP16	this study
loxP*dap1* -SYFP2/*pckmt* -Halo	*Dap1* is floxed and C-terminally tagged with SYFP2. *Pckmt* is C-terminally tagged with Halo	Inducible KO of *dap1* and analysis of its effect on PCKMT	this study
loxP*dap1* -SYFP2	Dap1 is floxed and C-terminally tagged with SYFP2	Inducible KO of *dap1* and plaque assays	this study
loxP*pckmt* -Halo/*pcr4* -SNAP	*Pckmt* is floxed and C-terminally tagged with Halo. Pcr4 is C-terminally tagged with SNAP	STED imaging anaysis of CGP and PCR4	this study
loxPcgp-SYFP2/pckmt-Halo	Cgp is floxed and C-terminally tagged with SYFP2. *Pckmt* is C-terminally tagged with Halo	Inducible KO of cgp and analysis of its effect on PCKMT	this study
loxP*frm1* -Halo/*pckmt* -SYFP2	*Frm1* is floxed and C-terminally tagged with Halo. *Pckmt* is C-terminally tagged with SYFP2	Inducible KO of *frm1* and analysis of its effect on PCKMT	this study
loxPcgp-Halo/iap3-SYFP2	*Cgp* is floxed and C-terminally tagged with Halo. *Iap3* is C-terminally tagged with SYFP2	Localisation and colocalisation analysis	this study
LoxP*pckmt* -Halo/210430-3HA/TLP1-mcherry	*Pckmt* is floxed and C-terminally tagged with Halo. *210430* is C-terminally tagged with 3HA. *Tlap1* is C-terminally tagged with mCherry	Colocalisation analysis	this study
loxP*cgp* -Halo/212780-SYFP2	*Cgp* is floxed and C-terminally tagged with Halo. 212780 is C-terminally tagged with SYFP2	Colocalisation analysis	this study
loxP210430-3HA	210430 is floxed and C-terminally tagged with 3HA	Inducible KO of 210430 and plaque assay	this study
LoxP*asaf1* -SYFP2	*Asaf1* is floxed and C-terminally tagged with SYFP2	Inducible KO of *asaf1* and phenotypic characterisation	this study
LoxP*asaf1* -3HA	*Asaf1* is floxed and C-terminally tagged with 3HA	Inducible KO of *asaf1*	this study
loxPasaf1-SYFP2/sfa2-3HA	*Asaf1* is floxed and C-terminally tagged with SYFP2. *Sfa2* is C-terminally tagged with 3HA	Inducible KO of *asaf1* and analysis its effect on SFA2	this study
loxP*asaf1* -3HA/*cgp* -Halo/*myoh* -SYFP2	*Asaf1* is floxed and C-terminally tagged with 3HA. Cgp is C-terminally tagged with Halo. MyoH is C-terminally tagged with SYFP2	Inducible KO of *asaf1* and analysis its effect on CGP and MyoH	this study
loxP*asaf1* -3HA/*cgp* -Halo/*rng2* -SYFP2	Asaf1 is floxed and C-terminally tagged with 3HA. Cgp is C-terminally tagged with Halo. RNG2 is C-terminally tagged with SYFP2	Inducible KO of *asaf1* and analysis its effect on CGP and RNG2	this study
loxP*asaf1* -3HA/*cgp* -Halo/*frm1* -SYFP2	Asaf1 is floxed and C-terminally tagged with 3HA. Cgp is C-terminally tagged with Halo. FRM1 is C-terminally tagged with SYFP2	Inducible KO of *asaf1* and analysis its effect on CGP and FRM1	this study
loxP*asaf1* -SYFP2/*imc1* -mCherry	*Asaf1* is floxed and C-terminally tagged with SYFP2.IMC1 is C-terminally tagged with mCherry	Inducible KO of *asaf1* and analysis its effect on IMC1 for live video microscopy	this study
loxP*asaf1* -SYFP2/*nuf2* -3HA	*Asaf1* is floxed and C-terminally tagged with SYFP2. *Nuf2* is C-terminally tagged with 3HA	Inducible KO of *asaf1* and analysis its effect on Nuf2	this study
loxP*asaf1* -SYFP2/*chromo1* -3HA	*Asaf1* is floxed and C-terminally tagged with SYFP2.*Chromo1* is C-terminally tagged with 3HA	Inducible KO of *asaf1* and analysis its effect on Chromo1	this study
loxP*asaf1* -SYFP2/*frm1* -Halo	*Asaf1* is floxed and C-terminally tagged with SYFP2.*Frm1* is C-terminally tagged with Halo	Inducible KO of *asaf1* and analysis its effect on FRM1	this study
loxP*asaf1* -SYFP2/*cgp* -Halo	*Asaf1* is floxed and C-terminally tagged with SYFP2. *Cgp* is C-terminally tagged with Halo	Inducible KO of *asaf1* and analysis its effect on CGP	this study
loxP*asaf1* -SYFP2/*myoh* -Halo	*Asaf1* is floxed and C-terminally tagged with SYFP2. *Myoh* is C-terminally tagged with Halo	Inducible KO of *asaf1* and analysis its effect on MyoH	this study
loxP*asaf1* -SYFP2/*rng2* -Halo	*Asaf1* is floxed and C-terminally tagged with SYFP2. *Rng2* is C-terminally tagged with Halo	Inducible KO of *asaf1* and analysis its effect on RNG2	this study
loxP*asaf1*- SYFP2/γ-*tubulin-3HA*	*Asaf1* is floxed and C-terminally tagged with SYFP2. γ-*tubulin* is C-terminally tagged with 3HA	Inducible KO of *asaf1* and analysis its effect on γ-tubulin	this study
loxP*cgp*- Halo/ *nd6* - SYFP2	Cgp is floxed and C-terminally tagged with Halo. *Nd6* is C-terminally tagged with SYFP2	Inducible KO of *asaf1* and analysis its effect on RNG4	this study

Table S5. Antibodies used for IFA in this study

Standard IFA

Primary antibodies

Name	Dilution	Origin / cat. Number
Rabbit α-GAP45	1/5000	Soldati's Lab
Mouse α-IMC1	1/2000	Ward's Lab
Mouse α-GFP	1/500	Merck, 11814460001
Rat α-HA	1/500	Merck, 11867423001
Camelid sdAb α-GFP-ATTO 488	1/500	Nano Tag Biotechnologies, N0304-At488-L
Camelid sdAb α-GFP-abberior star 635p	1/500	NanoTag Biotechnologies: N0304-Ab635P-L
Rabbit anti beta-tubulin	1/200	Morrissette's Lab
Streptavidin, Alexa Fluor 488 Conjugate	1/1000	Thermo Fisher Scientific, S11223
Mouse α-Centrin1	1/200	Merck, 04-1624
Rabbit anti- STAT6-P Tyr641	1/600	Cell Signaling 56554S
Mouse α-SAG1	1/2000	Lourido's Lab

Secondary antibodies

Name	Dilution	Origin/ cat. Number
Goat anti-Mouse IgG (H+L) Alexa Fluor 350	1/1000	Thermo Fisher Scientific, A11045
Goat anti-Rabbit IgG (H+L) Alexa Fluor 350	1/1000	Thermo Fisher Scientific, A11046
Goat anti-Mouse IgG (H+L) Alexa Fluor 488	1/1000	Thermo Fisher Scientific, A11001
Goat anti-Rat IgG (H+L) Alexa Fluor 488	1/1000	Thermo Fisher Scientific, A11006
Goat anti-Rabbit IgG (H+L) Alexa Fluor 488	1/1000-1/4000	Thermo Fisher Scientific, A32731
Goat anti-Rabbit IgG (H+L) Alexa Fluor 594	1/4000	Thermo Fisher Scientific, A11007
Donkey anti-Mouse IgG (H+L) Alexa Fluor 647	1/1000	Thermo Fisher Scientific, A31571
Chicken anti-Rat IgG (H+L) Alexa Fluor 647	1/1000	Thermo Fisher Scientific, A21472
Donkey anti-Rabbit IgG (H+L) Alexa Fluor 647	1/1000	Thermo Fisher Scientific, A31573
Abberior STAR 580, goat anti-mouse IgG	1/1000-1/3000	Abberior, ST580-1001-500UG
Abberior STAR 580, goat anti-rat IgG	1/1000	Abberior, ST580-1007-500UG
Abberior STAR 580, goat anti-rabbit IgG	1/1000	Abberior, ST580-1002-500UG
Abberior STAR 635P, goat anti-mouse IgG 500 µg	1/1000	Abberior, ST635P-1001-500UG
Abberior STAR 635P, goat anti-rat IgG 500 µg	1/1000	Abberior, ST635P-1007-500UG
Abberior STAR 635P, goat anti-rabbit IgG 500 µg	1/1000	Abberior, ST635P-1002-500UG
Goat anti-Mouse IgG - Atto 594	1/1000	Merck, 76085
Goat anti-Rabbit IgG - Atto 594	1/1000	Merck, 77671

Western Blots

Primary antibodies

Name	Dilution	Origin / cat. Number
IRDye 800CW α-Streptavidin	1/20000	LI-COR Biosciences, 926-32230
Rabbit α-aldolase	1/5000	Sibley' Lab
Mouse α-GFP	1/1000	Merck, 11814460001
Rat α-HA	1/1000	Merck, 11867423001

Secondary antibodies

Name	Dilution	Origin / cat. Number
IRDye680RD Donkey anti-Rabbit IgG	1/15000	LI-COR Biosciences, 926-68073
IRDye800CW Donkey anti-Mouse IgG	1/15000	LI-COR Biosciences, 926-32212
IRDye800CW Donkey anti-Rat IgG	1/15000	LI-COR Biosciences, 926-32219

ExM

Primary antibodies

Name		
rat α-HA	1/250	Merck, 11867423001
rabbit α-acetyl Tubulin (Lys40)	1/500	Merck ABT241
mouse α-IMC1	1/1000	Ward's Lab
mouse α-acetyl Tubulin	1/250	Merck, T6793
rabbit α-HA	1/500	Cell Signal Tech, 3724

Secondary antibodies

Name		
Hoechst 33342	1/25000	Thermo Fisher Scientific, 62249
Goat anti-Rat IgG (H+L) Alexa Fluor 488	1/1000	Thermo Fisher Scientific, A11006
Abberior STAR 635P, goat anti-rabbit IgG 500 µg	1/1000	Abberior ST635P-1002-500UG
Anti-Mouse IgG - Atto 594 antibody produced in goat	1/1000	Merck, 76085
Abberior STAR 635P, goat anti-mouse IgG 500 µg	1/1000	Abberior, ST635P-1001-500UG
Goat anti-Rabbit IgG (H+L) Alexa Fluor 488	1/1000	Thermo Fisher Scientific, A32731
