# Supplementary material for: An apical ring protein essential for conoid complex assembly and daughter cell formation in Toxoplasma gondii
Source: Nat Commun. 2025 Nov 28;16:10149. doi: 10.1038/s41467-025-65382-y (PMC12663574; doi:10.1038/s41467-025-65382-y)
Supplement: Supplementary file 2 — Description Of Additional Supplementary File [file 41467_2025_65382_MOESM2_ESM.pdf]

Description of Additional supplementary files

Supplementary Video 1:

CGP remains attached to the conoid throughout the lytic cycle. Videos showing CGP (in yellow) during replication, gliding, and invasion following egress. IMC1 is shown in magenta. The arrowhead indicates an invasion event after egress. Scale bar: 5 µm.

Supplementary Video 2:

Cryo-ET of WT phenotype parasite. Video of a reconstructed tomogram together with segmentation of the conoid complex of a WT T. gondii, as in Fig. 2c. Dark pink – PCRs, yellow – conoid, grey –APR, light pink – microtubules, dark blue – ICMTs, orange – membrane, violet – IMC

Supplementary Video 3:

Cryo-ET of cgp iKO phenotype parasite. Video of a reconstructed tomogram together with segmentation of the conoid complex of a cgp iKO T. gondii, as in Fig. 2c. Dark pink – PCRs, yellow – conoid, grey –APR, light pink – microtubules, dark blue – ICMTs, orange – membrane, violet – IMC.

Supplementary Video 4:

Videos of reconstructed tomograms. The selected top. Middle and bottom slices are shown in Fig. 2c, Supplementary Fig. 1c and Supplementary Fig.2. A – WT phenotype, w/o calcium ionophore; B – iKO phenotype, w/o calcium ionophore; C – WT phenotype, with calcium ionophore; D – iKO phenotype, with calcium ionophore. Scale bar – 200 nm.

Supplementary Video 5:

ASAF1 is present in daughter cells during early division stages. Video showing different z-stacks of images in Fig. 3b. Panels a–e corresponds to (1) – (5) in Fig. 3b. ASAF1 is shown in magenta, acetylated tubulin in green, and nuclei in blue. Arrows indicate spindle microtubules, while asterisks mark the spindle poles. Scale bar: 10 µm, and 5 µm for zoomed-in images.

Supplementary Video 6:

Supplementary video 6. Expansion microscopy show a lack of daughter cell formation in asaf1 iKO parasites. Video showing different z-stacks of images in Fig. 4b. White arrowheads indicate nascent conoid complex or the disrupted nascent conoid complex. Scale bar: 10 µm

Supplementary Video 7:

Absence of ASAF1 leads to failure in parasite division. Video showing induced and non-induced loxPasaf1-SYFP2 KO parasite replication marked either with IMC1 (top row) or tubulin (bottom row). The videos correspond to images in Supplementary Fig. 12. Scale bar: 5 µm.
