# Supplementary material for: An apical ring protein essential for conoid complex assembly and daughter cell formation in Toxoplasma gondii
Source: Nat Commun. 2025 Nov 28;16:10149. doi: 10.1038/s41467-025-65382-y (PMC12663574; doi:10.1038/s41467-025-65382-y)
Supplement: Supplementary file 10 — Reporting summary [file 41467_2025_65382_MOESM10_ESM.pdf]

nature portfolio

Corresponding author(s): Li, W
Jimenez-Ruiz, E
Meissner, M

Last updated by author(s): Aug 15, 2025

Reporting Summary

Nature Portfolio wishes to improve the reproducibility of the work that we publish. This form provides structure for consistency and transparency in reporting. For further information on Nature Portfolio policies, see our Editorial Policies and the Editorial Policy Checklist.

Statistics

For all statistical analyses, confirm that the following items are present in the figure legend, table legend, main text, or Methods section.

n/a	Confirmed	
☐	☒	The exact sample size (*n*) for each experimental group/condition, given as a discrete number and unit of measurement
☐	☒	A statement on whether measurements were taken from distinct samples or whether the same sample was measured repeatedly
☐	☒	The statistical test(s) used AND whether they are one- or two-sided<br>*Only common tests should be described solely by name; describe more complex techniques in the Methods section.*
☒	☐	A description of all covariates tested
☒	☐	A description of any assumptions or corrections, such as tests of normality and adjustment for multiple comparisons
☐	☒	A full description of the statistical parameters including central tendency (e.g. means) or other basic estimates (e.g. regression coefficient) AND variation (e.g. standard deviation) or associated estimates of uncertainty (e.g. confidence intervals)
☐	☒	For null hypothesis testing, the test statistic (e.g. *F*, *t*, *r*) with confidence intervals, effect sizes, degrees of freedom and *P* value noted<br>*Give P values as exact values whenever suitable.*
☒	☐	For Bayesian analysis, information on the choice of priors and Markov chain Monte Carlo settings
☒	☐	For hierarchical and complex designs, identification of the appropriate level for tests and full reporting of outcomes
☒	☐	Estimates of effect sizes (e.g. Cohen's *d*, Pearson's *r*), indicating how they were calculated

Our web collection on statistics for biologists contains articles on many of the points above.

Software and code

Policy information about availability of computer code

Data collection	LasX software from Leica and Instructor from Abberior was used to obtain parasite imaging data and LI-COR Image Studio Software for WB images. ApE and BioEdit open source software for in-silico cloning and sequences analysis. This information is added to the materials and methods section with version numbers
Data analysis	Graphpad Prism 8.2.1. Fiji (ImageJ), Excel, Huygens essential and Icy software were used for analysing the data. ApE and BioEdit open source software for in-silico cloning and sequences analysis. All these software packages are described in materials and methods

For manuscripts utilizing custom algorithms or software that are central to the research but not yet described in published literature, software must be made available to editors and reviewers. We strongly encourage code deposition in a community repository (e.g. GitHub). See the Nature Portfolio guidelines for submitting code & software for further information.

Data

Policy information about availability of data

All manuscripts must include a data availability statement. This statement should provide the following information, where applicable:

- Accession codes, unique identifiers, or web links for publicly available datasets
- A description of any restrictions on data availability
- For clinical datasets or third party data, please ensure that the statement adheres to our policy

The mass spectrometry proteomics data have been deposited to the ProteomeXchange Consortium via the partner repository 58. with the dataset identifier

PXD053452. All parasite strains and imaging data generated for this paper are available from the authors on reasonable request. The source data are provided as a Source Data file.

Research involving human participants, their data, or biological material

Policy information about studies with human participants or human data. See also policy information about sex, gender (identity/presentation), and sexual orientation and race, ethnicity and racism.

Reporting on sex and gender	Not applicable
Reporting on race, ethnicity, or other socially relevant groupings	Not applicable
Population characteristics	Not applicable
Recruitment	Not applicable
Ethics oversight	Not applicable

Note that full information on the approval of the study protocol must also be provided in the manuscript.

Field-specific reporting

Please select the one below that is the best fit for your research. If you are not sure, read the appropriate sections before making your selection.

☒ Life sciences ☐ Behavioural & social sciences ☐ Ecological, evolutionary & environmental sciences

For a reference copy of the document with all sections, see nature.com/documents/nr-reporting-summary-flat.pdf

Life sciences study design

All studies must disclose on these points even when the disclosure is negative.

Sample size	All quantifications had a minimum n of 100 parasites or vacuoles per condition and replicate. We performed 3 technical replicates in each biological replicate (n = 3).
Data exclusions	No data was excluded from this study
Replication	All quantifications were made in triplicates successfully.
Randomization	Not applicable
Blinding	We did no allocate the groups blindly but we quantify the phenotypes observed and related the results to wildtype parasites to avoid vias of the data.

Reporting for specific materials, systems and methods

We require information from authors about some types of materials, experimental systems and methods used in many studies. Here, indicate whether each material, system or method listed is relevant to your study. If you are not sure if a list item applies to your research, read the appropriate section before selecting a response.

Materials & experimental systems

n/a	Involved in the study	
☐	☒	Antibodies
☐	☒	Eukaryotic cell lines
☒	☐	Palaeontology and archaeology
☒	☐	Animals and other organisms
☒	☐	Clinical data
☒	☐	Dual use research of concern
☐	☐	Plants

Methods

n/a	Involved in the study	
☒	☐	ChIP-seq
☒	☐	Flow cytometry
☒	☐	MRI-based neuroimaging

Antibodies

Antibodies used	Rabbit α-GAP45 (polyclonal supplied by Soldati lab; Egarter et al., 2014), mouse α-IMC1 (hybridoma, supplied by Ward lab; Harding et al., 2016), mouse α-GFP (Roche, 11841460001), rat α-HA (Roche, 1187431001), α-GFP-ATTO 488 (Nano Tag Biotechnologies, N0304-At488-L; Li et al., 2022).<br>Secondary antibodies: Alexa Fluor 350 (Thermo Fisher Scientific, A11045, A11046), Alexa Fluor 488 (Thermo Fisher Scientific, A11001, A11006, A32731) or Abberior STAR 580 (Abberior,ST580-1001-500UG, ST580-1002-500UG) or Abberior STAR 635P (Abberior, ST635P-1001-500UG, ST635P-1002-500UG).
Validation	All non-commercial antibodies are regularly used in our lab and have been published /validated in previous studies (see references above). No novel antibodies were generated in this study .<br>The antibodies that recognise tags like GFP and HA are commonly used and validated by the manufacturer (links to respective validation provided): mouse α-GFP (Roche, 11841460001; https://www.sigmaaldrich.com/DE/en/product/roche/11814460001), rat α-HA (Roche, 1187431001; https://www.sigmaaldrich.com/DE/en/product/roche/roahaha), α-GFP-ATTO 488 (Nano Tag Biotechnologies, N0304-At488-L; https://sysy.com/product/N0304-At488-L).

Eukaryotic cell lines

Policy information about cell lines and Sex and Gender in Research

Cell line source(s)	Human Foreskin Fibroblasts (HFF) obtained from ATCC (ATCC-SCRC-1041). Toxoplasma gondii lines derived from RHdeltaHxprt and RHdeltaHxprt deltaKu80 (Huynh and Carruthers, 2009)
Authentication	Transgenic parasites and knockout parasites were validated using PCR and sequencing.
Mycoplasma contamination	HFF are tested regularly for mycoplasm and are negative.
Commonly misidentified lines<br>(See ICLAC register)	No commonly misidentified cell lines were used in the study

Plants

Seed stocks	N/A
Novel plant genotypes	N/A
Authentication	N/A
